# Economic evaluations of onchocerciasis interventions: a systematic review and research needs

**DOI:** 10.1111/tmi.13241

**Published:** 2019-05-09

**Authors:** Hugo C. Turner, Martin Walker, Sébastien D. S. Pion, Deborah A. McFarland, Donald A. P. Bundy, María‐Gloria Basáñez

**Affiliations:** ^1^ Oxford University Clinical Research Unit Wellcome Africa Asia Programme Ho Chi Minh City Vietnam; ^2^ Centre for Tropical Medicine and Global Health Nuffield Department of Medicine University of Oxford Oxford UK; ^3^ London Centre for Neglected Tropical Disease Research Department of Pathobiology and Population Sciences Royal Veterinary College Hatfield UK; ^4^ London Centre for Neglected Tropical Disease Research Department of Infectious Disease Epidemiology School of Public Health Imperial College London London UK; ^5^ Institut de Recherche pour le Développement UMI 233‐INSERM U1175‐Montpellier University Montpellier France; ^6^ Rollins School of Public Health Emory University Atlanta GA USA; ^7^ London School of Hygiene and Tropical Medicine London UK; ^8^ MRC Centre for Global Infectious Disease Analysis Department of Infectious Disease Epidemiology School of Public Health Imperial College London London UK

**Keywords:** onchocerciasis, river blindness, economic evaluations, cost effectiveness, cost‐benefit analyses, cost, elimination, health economics, onchocercose, cécité des rivières, évaluations économiques, rapport coût‐efficacité, analyses coût‐bénéfice, coût, élimination, économie de la santé

## Abstract

**Objective:**

To provide a systematic review of economic evaluations that has been conducted for onchocerciasis interventions, to summarise current key knowledge and to identify research gaps.

**Method:**

A systematic review of the literature was conducted on the 8th of August 2018 using the PubMed (MEDLINE) and ISI Web of Science electronic databases. No date or language stipulations were applied to the searches.

**Results:**

We identified 14 primary studies reporting the results of economic evaluations of onchocerciasis interventions, seven of which were cost‐effectiveness analyses. The studies identified used a variety of different approaches to estimate the costs of the investigated interventions/programmes. Originally, the studies only quantified the benefits associated with preventing blindness. Gradually, methods improved and also captured onchocerciasis‐associated skin disease. Studies found that eliminating onchocerciasis would generate billions in economic benefits. The majority of the cost‐effectiveness analyses evaluated annual mass drug administration (MDA). The estimated cost per disability‐adjusted life year (DALY) averted of annual MDA varies between US$3 and US$30 (cost year variable).

**Conclusions:**

The cost benefit and cost effectiveness of onchocerciasis interventions have consistently been found to be very favourable. This finding provides strong evidential support for the ongoing efforts to eliminate onchocerciasis from endemic areas. Although these results are very promising, there are several important research gaps that need to be addressed as we move towards the 2020 milestones and beyond.

## Abstract (fr)

**Objectif:**

Fournir une analyse systématique des évaluations économiques réalisées pour les interventions contre l'onchocercose, résumer les principales connaissances actuelles et identifier les lacunes de la recherche.

**Méthode:**

Une revue systématique de la littérature a été menée le 8 août 2018 en utilisant les bases de données électroniques PubMed (Medline) et ISI Web of Science. Aucune indication de date ou de langue n'a été appliquée aux recherches.

**Résultats:**

Nous avons identifié 14 études principales rapportant sur les résultats d’évaluations économiques d'interventions contre l'onchocercose, dont 7 étaient des analyses coût‐efficacité. Les études identifiées ont utilisé diverses approches pour estimer les coûts des interventions/programmes étudiés. A l'origine, les études ne mesuraient que les avantages associés à la prévention de la cécité. Progressivement, les méthodes se sont améliorées et ont également capturé les maladies de la peau associées à l'onchocercose. Les études ont montré que l’élimination de l'onchocercose générerait des milliards de bénéfices économiques. La majorité des analyses coût‐efficacité ont évalué l'administration annuelle en masse de médicaments (AMD). Le coût estimé par année de vie ajustée par rapport à l'incapacité (DALY) corrigé pour l’AMD annuelle varie entre 3 et 30 USD (variable de l'année de coût).

ConclusionsLe rapport coût‐efficacité et la rentabilité des interventions contre l'onchocercose se sont toujours avérés très favorables. Cette constatation fournit un solide appui probant aux efforts en cours pour éliminer l'onchocercose des zones d'endémie. Bien que ces résultats soient très prometteurs, plusieurs lacunes importantes en matière de recherche doivent être comblées à mesure que nous nous approchons des étapes clés pour 2020 et au‐delà.

## Introduction

Human onchocerciasis, also known as ‘river blindness’, is a parasitic infection caused by the filarial nematode *Onchocerca volvulus*. It is transmitted by the bites of *Simulium* blackflies. Of the 120 million people at risk, 99% live in sub‐Saharan Africa, although the disease has also been endemic in six countries of Latin America and is present in Yemen 1. Symptoms can include severe itching, disfiguring skin conditions, visual impairment, and permanent blindness 1. Onchocerciasis is the world's second leading infectious cause of blindness after trachoma 2. Onchocerciasis can also cause excess mortality 3, 4, 5 and is associated with epilepsy, nodding syndrome and hyposexual dwarfism (Nakalanga syndrome) 6, 7, 8.

During the last 40 years, there has been a remarkable expansion of onchocerciasis control programmes worldwide 9, 10, 11, 12, 13, 14, summarised in Box 1. These programmes have had a large impact on reducing onchocerciasis as a public health problem. Initially, control programmes (i.e. the Onchocerciasis Control Programme in West Africa (OCP)) only used large‐scale vector control as the drugs available at that time were too toxic for large‐scale use. This changed in 1987, when ivermectin (produced under the brand name Mectizan^®^), a drug suitable for mass treatment, was registered for human use against onchocerciasis, and large‐scale chemotherapeutic control programmes became feasible 11.

Box 1 **Summary of the control programmes**

**The Onchocerciasis Control Programme in West Africa (OCP, 1974–2002):** The OCP was launched in 1974–1975, and originally covered a core area in seven countries of West Africa 11. However, by 1990 the OCP had expanded its operations to include larger zones and four additional countries following the southern and western extensions 10, 11. From 1974 to 1988 the OCP focused on a strategy of weekly aerial larviciding of blackfly breeding sites. In 1987 ivermectin was registered for human use against onchocerciasis, and due to the suitability of this drug for mass treatment, large‐scale chemotherapeutic control programmes became feasible 10. Large‐scale mass drug administration (MDA) of ivermectin began in the OCP regions in the late 1980s, initially administered by mobile teams of paid, local health professionals 10.

**African Programme for Onchocerciasis Control (APOC, 1995–2015):** The APOC was initiated in 1995 including 19 African countries 11, 149. The programme pioneered a community‐directed treatment approach, within which the local communities rather than health services directed the treatment process; the treatments were delivered by volunteer community‐directed distributors (CDDs) 149, 150, 151. APOC gradually expanded and by 2014 it had a network of over 699 656 volunteer CDDs 151. When the programme concluded at the end of 2015 it was supporting onchocerciasis control and elimination activities in 31 African countries (including the 19 original signatories of the Memorandum, South Sudan, and the 11 ex‐OCP participating countries) 149.

**The Onchocerciasis Elimination Program for the Americas (OEPA, 2002–present):** The OEPA started in 1992 in six countries of the Americas (across 13 discrete foci) 11, 12. The strategy is based on the biannual (twice a year) distribution of ivermectin, to all endemic communities (covering at least 85% of the eligible population) 12. As of December 2016, a total of four countries have successfully completed the World Health Organization process for verification of elimination 152.

In 1987, Merck & Co. committed to supply ivermectin to onchocerciasis endemic countries ‘as much as necessary for as long as necessary’ 15, 16. Over 7.8 billion ivermectin tablets have been donated for the treatment of onchocerciasis and lymphatic filariasis 17. This unprecedented donation allowed onchocerciasis control in endemic areas where vector control was not feasible or too expensive to sustain and ivermectin began to be distributed in the late 1980s. Initially, mobile teams of paid, local health professionals were used to distribute ivermectin; however, this was costly 18, and programmes mostly switched to using volunteer distributors and community‐directed approaches (Box 1).

Motivated by the successful elimination of the infection in some foci of Mali and Senegal 19, 20, there was a shift in onchocerciasis control policy in Africa, with the aim of programmes changing from elimination as a public health problem to elimination of transmission and ultimately infection. In 2012, the Joint Action Forum of the African Programme for Onchocerciasis Control (APOC), chaired by WHO and the ministers of health of endemic countries, set the target at elimination in 80% of African countries by 2025 21. WHO's roadmap on neglected tropical diseases (NTDs) also included goals for the elimination in several African countries by 2020 22.

The coverage of NTD mass drug administration (MDA) programmes has notably expanded over the last 10 years. In 2017, 1.762 billion treatments were delivered worldwide 23. NTD control programmes are becoming increasingly integrated, moving towards targeting multiple diseases within a multipronged programme 24, 25. In May 2016, the WHO launched the Expanded Special Project for Elimination of Neglected Tropical Diseases (ESPEN), a five‐year project to provide national NTD programmes with technical and fundraising support to help them accelerate the control and elimination of NTDs amenable to preventive chemotherapy, namely onchocerciasis, lymphatic filariasis, schistosomiasis, soil‐transmitted helminthiases and trachoma 26. The integrated and cross‐sectorial response to NTD control is relevant to the Sustainable Development Goals, including among others Universal Health Coverage, Water Sanitation and Hygiene (WASH) initiatives, global partnerships, alleviating poverty and hunger and improving education and economic growth 27.

The aim of this paper was to provide a systematic review of economic evaluations that have been conducted for onchocerciasis interventions, to summarise current key knowledge and to identify research gaps in this area.

## Methods

### Search strategy

**Figure 1:**
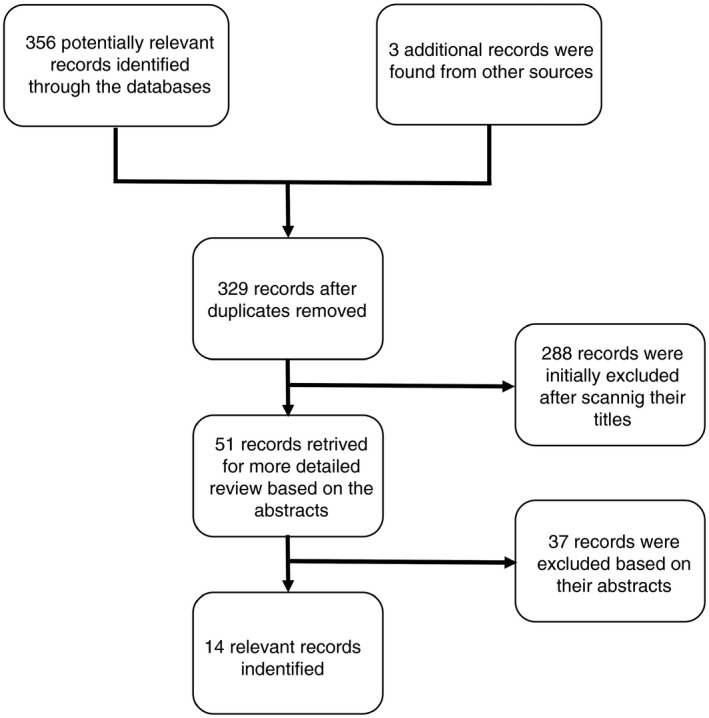
Decision tree outlining the inclusion and exclusion of the identified studies. Some studies reported both cost‐benefit and cost‐effectiveness estimates. A PRISMA checklist is provided in Appendix S1.

A systematic review of the literature was conducted on the 8th of August 2018 using the PubMed (MEDLINE) and ISI Web of Science electronic databases. Variants of the following search terms were used to find relevant papers: river blindness, onchocerciasis, cost(s), cost‐benefit, cost‐effectiveness, economic(s), economic evaluation. No date or language stipulations were applied to the searches. A more detailed summary of the search terms and the PRISMA checklist are supplied in Appendix S1. The titles and abstracts of all the identified papers were examined initially for relevance and then the bibliographies of papers suitable for inclusion were scanned for studies not originally retrieved from the databases. The full selection process is outlined in Figure 1. Studies relating to cost recovery and willingness to pay were not explicitly included as an outcome of the systematic literature search (but are referenced within the review where relevant).

## Results and discussion

We identified 14 primary studies reporting the results of economic evaluations of onchocerciasis interventions, which are described in Tables 1 and 2. It is important to note that both the OCP and APOC expanded over time (Box 1) and it was not always clear which specific countries/areas were being included in the different analyses.

**Table 1:** Summary of the identified cost‐benefit analyses and estimates of the economic benefits of onchocerciasis interventions

Source	Setting and time period of the intervention	Time horizon for the benefits	Discount rate	Cost year	Cost of the intervention	Total economic benefit^b^	Net present value	Internal rate of return
Benton & Skinner 67	OCP (1974–2004)	1974–2023	5–10%	1985 US$	US$140 million (10% discount rate) to US$231 million (5% discount rate). US$437 million when not discounted. Financial costs from the programmes perceptive. Details not specified.	US$148 million (10% discount rate) to US$543 million (5% discount rate).	US$8 million (10% discount rate) to US$312 million (5% discount rate).	11–13%
Kim & Benton 58	OCP (1974–2002)	1974–2012	3–10%	1987 US$	US$571.2 million (appears to be pre‐discounting). Financial costs from the programmes perceptive. Based on actual and projected OCP expenditure.	Not stated.	US$485 million (10% discount rate) to US$3729 million (3% discount rate).	20%
McFarland & Murray 124, a	OCP (10‐year project time period)	1974–2023	5%	Not available	US$195.5 million (not discounted). Details not available.	Not available.	US$8 million.	‐
Benton 125	APOC (1996–2007)	1996–2017	10%	1996 US$	US$131.2 million (appears to be pre‐discounting). Financial costs from the healthcare providers perceptive. Details not specified.	Not stated.	US$53.7 million.	17%
Haddix 69, a	APOC (1996–2007)	1996–2017	3–10%	1996 US$	US$108.5 million (unclear if discounted). Details not available.	Not available.	US$87.6 million (10% discount rate) to US$307.4 million (3% discount rate).	24%
Kim *et al*. 60	Potential benefits of achieving elimination scenarios in Africa	2013–2045	3%	2013 US$	NA	Compared with the control scenario, the Elim I and II scenarios^c^ would generate US$5.96 (2.53–7.28) billion and US$6.46 (2.83–8.09) billion in economic benefits respectively.	NA	NA
Redekop *et al*. 59	Potential economic benefits of achieving the WHO 2020 targets	2011–2030	3%	2005 US$	NA	US$3.3 (2.4–5.11) billion.	NA	NA
Turner *et al*. 95	Potential impact of moxidectin on onchocerciasis elimination in African savannah settings^d^	50 years	3%	2012 US$	Moxidectin distribution was assumed to cost the same as that for ivermectin. The relative total cost of using moxidectin *vs*. ivermectin was considered for different programmatic scenarios^d^. Used the healthcare providers perspective (not including the value of the donated drugs). Based on a costing study in Ghana 44.	Annual moxidectin treatment would achieve similar reductions in programme duration as using biannual ivermectin treatment. If the moxidectin tablets were donated its use would lead to substantial in‐country cost savings.	NA	NA

APOC, African Programme for Onchocerciasis Control; NA, not applicable; OCP, Onchocerciasis Control Programme in West Africa; pOTTIS, provisional operational thresholds for treatment interruption followed by surveillance.

a Information for this study was taken from Waters *et al*. 18.

b The estimated economic benefits are outlined in further detail in Table 3.

c The Control, Elim I and Elim II scenarios are described in Kim *et al*. 99.

d Assumed that MDA would be stopped (determining the programme duration) once the pOTTIS would have been achieved (defined as the modelled microfilarial prevalence being <1.4%, measured just before the next treatment round).

**Table 2:** Summary of the identified cost‐effectiveness analyses of onchocerciasis interventions

Study	Setting and time period of the intervention	Time horizon for the benefits	Discount rate	Cost year	Cost of the intervention	Effectiveness	Cost‐effectiveness ratio
McFarland & Murray 124, a	OCP	‐	‐	Not available	US$19.5 million per year. Details not available.	640 000 DALYs lost annually in absence of control. Details not available.	If all of the onchocerciasis related DALYs were eliminated, the programme would cost US$30.47 per DALY averted.
Prescott *et al*. 126 and Prost & Prescott 127	OCP ‐ Upper Volta (now Burkina Faso) (1975–1994)	1975–1994	10%	1977 US$	US$22.1 million. Financial costs from the programmes perceptive. Based on actual and projected OCP expenditure.	147 294 healthy life‐years added. Based on the estimated number of blindness cases prevented. Assumed that one blindness case results in 23 years healthy life lost in hyperendemic and 20 in mesoendemic areas. Assumed that blindness is associated with a disability weight of 1.	US$150 per healthy life‐year added. When not discounting the effectiveness, the results changed to US$20 per healthy life‐year added.
Evans *et al*. 71	OCP ‐ Burkina Faso (1974–1997)	1974–1997	10% (but varied between 3–15%)	1984 US$	US$115 million (appears to be pre‐discounting). Financial costs from the programmes perceptive. Based on actual and projected OCP expenditure.	21 567 healthy life‐years added. Based on the estimated number of blindness cases prevented. Assumed that one blindness case results in 18.7 years healthy life lost in hyperendemic and 15 in mesoendemic areas. Assumed that blindness is associated with a disability weight of 0.5.	US$2119 per healthy life‐year added (10% discount rate). When using a 3% discount rate the results changed to US$1028 per healthy life‐year added.
Benton 125	APOC (1996–2007)	1996–2017	3%	1996 US$	US$131.2 million (not clear if the costs were discounted). Financial costs from the healthcare providers perceptive. Details not specified.	9 788 304 health life‐years added. Based on the estimated number of blindness cases prevented. Assumed each case of blindness results in 20 discounted healthy life‐years lost. Assumed that blindness is associated with a disability weight of 1.	US$13.4 per healthy life‐year added.
Coffeng *et al*. 30	APOC (1995–2015)	1995–2015	0%	Nominal values	US$478 million. Financial costs from the programmes perceptive. Based on APOC financial reports for the World Bank.	17.4 million DALYs averted (not discounted). Estimated using a dynamic transmission model (ONCHOSIM). Used the GBD 2004 disability weights (Table 5).	US$27 per DALY averted.
Remme *et al*. 63	APOC (over 15 years)	Over a 25‐year period	Unclear	Not stated	US$209 million. Financial cost from the healthcare providers perceptive. Source not stated.	At least 26 million DALYs averted. Estimated using a back of the envelope calculation. Details on the DALY calculation/weights not given.	Approximately US$7 per DALY averted.
Turner *et al*. 73	Annual MDA in an African savannah setting (up to 50 years)^b^^*,*^ c	50 years	3%	2012 US$	US$0.55–1.07 million per 100 000 – depending on the assumed endemicity level^c^. Assumed that once the pOTTIS was achieved, MDA would be stopped^b^. Economic cost from the healthcare providers perspective (not including the value of the donated ivermectin). Based on a costing study in Ghana 44.	37 858–331 632 DALYs averted per 100 000 –depending on the assumed endemicity level^c^. Estimated using a dynamic transmission model (EPIONCHO). Used the GBD 2004 disability weights (Table 5). Included the excess mortality associated with heavy infections 83.	US$3–15 per DALY averted – depending on the assumed endemicity level^c^. Results changed to US$29–133 per DALY averted when including the additional economic value of the donated ivermectin. If elimination not achieved the results for the lowest endemicity setting would change from US$15 to US$28 per DALY averted.
Turner *et al*. 73	Biannual MDA in an African savannah setting (up to 50 years)^b^^*,*^ c	50 years	3%	2012 US$	US$0.63–1.20 million per 100 000 – depending the assumed endemicity level^c^. Incremental to annual treatment: US$0.07–0.13 million per 100 000. Assumed that once the pOTTIS was achieved, MDA would be stopped^b^. Economic cost from the healthcare providers perspective (not including the value of the donated ivermectin). Based on a costing study in Ghana 44.	38 585–342 229 DALYs averted per 100 000 –depending the assumed endemicity level^c^. Incremental to annual treatment: 727–10 597 per 100 000. Estimated using a dynamic transmission model (EPIONCHO). Used the GBD 2004 disability weights (Table 5). Included the excess mortality associated with heavy infections 83.	Incremental cost‐effectiveness ratio: US$12–100 per incremental DALY averted – depending on the assumed endemicity level^c^. Results changed to US$334–2674 per incremental DALY averted when including the additional economic value of the donated ivermectin.

APOC, African Programme for Onchocerciasis Control; DALY, disability‐adjusted life years; MDA, mass drug administration; Nominal cost, values have not been adjusted for inflation; OCP, Onchocerciasis Control Programme in West Africa; pOTTIS, provisional operational thresholds for treatment interruption followed by surveillance.

a Information for this study was taken from Waters *et al*. 18.

b Assumed that MDA would be stopped (determining the programme duration and its total cost) once the pOTTIS would have been achieved (defined as the modelled microfilarial prevalence being <1.4%, measured just before the next treatment round).

c Three different endemicity levels were explored (ranging between 40–80% microfilarial prevalence).

### Estimated cost of onchocerciasis interventions

A key component of any cost‐benefit or cost‐effectiveness analysis (Box 2) of an intervention is its estimated cost. The studies identified used a variety of different approaches to estimate the costs of the investigated interventions/programmes. Many of the studies based their costs on programme budgets/reports and very few were based on comprehensive costing studies/approaches. There was variation in what types of costs were included within the analyses.

Box 2 **Glossary**

**Cost‐benefit analysis:** A type of economic evaluation which compares the cost of an intervention to its monetary benefits. The results are typically expressed as an internal rate of return or net present value.

**Cost‐effectiveness analysis:** A type of economic evaluation in which the cost of an intervention is compared to the quantity of a single non‐monetary effectiveness measure (such as the number of deaths or cases averted). This avoids the issues associated with monetising the benefits of healthcare interventions. The results are expressed as a cost per unit of outcome (see cost‐effectiveness ratio).

**Cost‐effectiveness ratio:** A statistic used to summarise the cost‐effectiveness of an intervention. It is calculated by dividing the cost of an intervention by its effectiveness measure, such as a cost per disability‐adjusted life year (DALY) averted or healthy life year gained. An **incremental cost‐effectiveness ratio** is calculated by dividing the difference in costs by the difference in effectiveness outcomes of two alternative options (it summaries the ‘extra cost per additional unit of effect gained’).

**Community‐directed distributors (CDDs):** Also referred to as community drug distributors, these are volunteers selected by their communities to distribute treatment.

**Disability‐adjusted life years (DALYs):** A measure of disease burden that is calculated as the sum of the years of life lost due to premature mortality and the years of healthy life lost due to disability. The number of years of healthy life lost due to disability is calculated using a disability weight factor (between 0 and 1) that reflects the severity of the disease/disability. One DALY can be thought of as one year of ‘healthy’ life lost.

**Economic costs (opportunity costs):** These define the cost of a resource as its value in its next best alternative use (also known as an opportunity cost). This is a broader conceptualisation of a resource's value than its financial cost, as it recognises that using a resource makes it unavailable for productive use elsewhere. The rationale behind economic costs is that they are intended to represent the full value of all the resources used for an intervention, and they account for the fact that resources can have a value that is not (fully) captured by their financial costs (such as the ‘free’ use of building space provided by Ministries of Health, and the unpaid time devoted to mass drug administration by volunteer CDDs). This is particularly important when considering issues related to the sustainability and replicability of interventions.

**Economies of scale:** The reduction in the average cost per unit resulting from increased production/output; in this case, the reduction in the cost per treatment as a result of increasing the number of people treated.

**Economies of scope**: The reduction in the average cost per unit resulting when providing multiple goods/services jointly; in this case, the reduction in the cost per treatment when delivering more than one intervention at once (e.g. integrated control programmes or using the CDD platform to deliver more than a single intervention). Examples include administering treatment for both schistosomiasis and soil‐transmitted helminthiases within the same programme (instead of by separate vertical programmes).

**Financial costs:** The actual expenditure (i.e. the amount paid) for the goods, resources and services that are purchased.

**Friction cost approach:** The approach that takes the employer's perspective for valuing lost productivity, and therefore only counts as lost, the hours not worked by a sick employee before another employee takes over the work 65. It is based on the assumption that an ill individual will eventually be replaced by another healthy worker – therefore, the initial productivity levels are restored after this ‘friction period’.

**Human capital approach:** The approach that takes the patient's perspective for valuing lost productivity and therefore counts all the work they miss, as a productivity loss. With this approach, all potential production not performed by an individual because of morbidity or premature mortality is counted as a production loss 65.

**Indirect costs (productivity costs):** Indirect cost represents the value of productivity losses that result from illness, treatment, or premature death.

**Internal rate of return (IRR):** The discount rate applied to the monetised benefits and costs of an intervention, that makes its net present value equal to zero. Also known as the economic rate of return (ERR).

**Mass drug administration (MDA):** The large‐scale distribution of drugs to eligible people within populations at risk of infection, irrespective of current individual infection status, i.e. without the need for screening for or diagnosing infection prior to each treatment round.

**Net present value (NPV):** The difference between an intervention's monetised benefits and its cost. A positive NPV is an indicator of a successful investment.

**Perspective:** The viewpoint from which the intervention's costs and consequences are evaluated. When adopting the healthcare providers perspective, the costs falling outside the healthcare sector are ignored. In contrast, when adopting the societal perspective, all relevant cost categories should be included, including those incurred by the patients.

**Time horizon:** The time horizon for the analysis; the duration over which outcomes and costs are calculated.

The estimated intervention costs tended to be higher for the studies evaluating OCP activities. This is likely because the control measures used by the OCP (vector control and mass treatment delivered via mobile teams) were more expensive than the community‐directed treatment approach subsequently used by other programmes 28 (Box 1). The nominal financial cost (i.e. not adjusted for inflation or discounted) of the OCP (1974–2002) was just under US$1 billion 29 whereas the projected cost of the larger APOC (1995–2015) was US$478 million 30.

Often it was difficult to compare the reported costs from the different studies, as it was not always clear how costs were estimated, what activities were costed, and whether reported values were discounted or not. Some studies appeared only to adjust the costs for inflation and not discount them (Box 3).

Box 3 **Discounting**

Healthcare interventions typically incur costs and generate health outcomes over a number of years. However, society does not place an equal value on costs or health outcomes that occur now compared to those that occur in the future. This is because there is an opportunity cost to spending money (as it could be invested to yield returns) and a desire to have benefits now rather than in the future. Economic evaluations therefore need to weight differently costs and health outcomes that occur in the future.

Discounting is the process used to convert costs or health outcomes occurring in the future into a present value 153, 154, 155. It makes costs and benefits occurring in the future worth less than those in the present. This allows the comparison of the costs and outcomes occurring over different time periods. The discount rate determines the strength of the time preference – the higher the discount rate the lower the value placed on future costs/outcomes. Note that adjusting for inflation (which accounts for the fact that the purchasing power of a currency changes over time) is not the same as discounting.

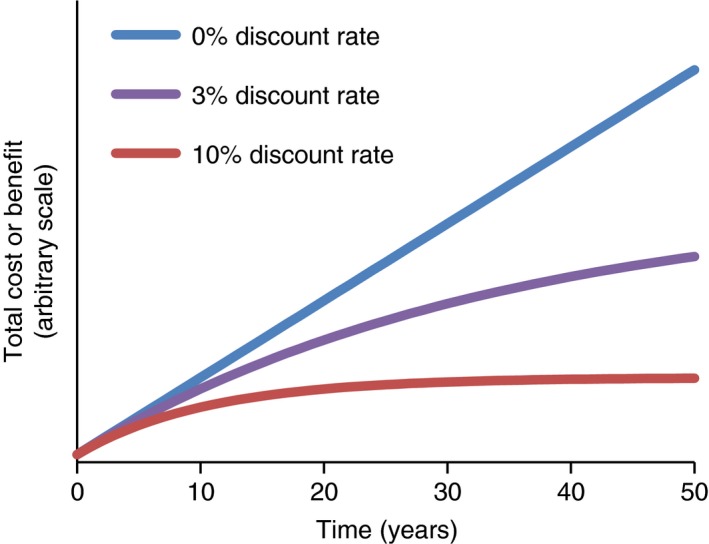

When different analyses have used different discount rates, it makes the results harder to compare them directly. The WHO's guide to cost‐effectiveness analysis recommends using a 3% discount rate for both costs and health outcomes (and testing the sensitivity of the results to using a 0% discount rate for health effects and a 6% discount rate for costs within the sensitivity analysis) 50. These recommendations have engendered greater consistency in the use of discount rates. However, although using the 3% discount rate has become the standard, there is still debate within the field, particularly on discounting of health effects 154, 155.

The studies evaluating programmes using community‐based mass treatment generally assumed delivery costs of around US$0.50 per treatment. This is consistent with the findings of a systematic review by Keating *et al*. 31, in which the average of identified onchocerciasis‐specific cost estimates was US$0.46 per treatment, as well as with recent MDA cost benchmarks estimated by the WHO 32, 33. However, it is important to note that the costs of MDA delivery vary across different settings. An important driver in this variation is the size of the targeted population 34, 35, 36. This is because MDA programmes generate economies of scale; as the number of people treated increases, the cost per treatment tends to decrease 37, 38. NTD control programmes have also become increasingly integrated and instead of using separate disease‐specific programmes they now often target multiple diseases within one programme. This can result in economies of scope, reducing the overall cost of the NTD interventions 31, 37, 38, 39, 40, 41, 42, 43. Most of the studies identified did not consider the potential impact of economies of scale or scope on the costs of the interventions within their analyses.

There are very few studies that have investigated the costs of alternative onchocerciasis interventions to annual community‐directed MDA (with ivermectin) 32. Turner *et al*. 44 found that the yearly cost of a community‐directed ivermectin treatment programme increases by 50–60% when increasing the treatment frequency from once to twice a year.

Several studies also considered the potential for cost‐recovery/cost‐sharing, where the communities contribute financially to the cost of the programme 28, 45, 46, 47 and the participants’ willingness to pay for ivermectin treatment 48, 49.

#### Economic costs vs. financial costs

The majority of the studies considered only the financial costs of the intervention. However, when performing economic evaluations of healthcare interventions, it is typically recommended to use ‘economic costs’ 50, 51 (Box 2). These conceptualise costs in a broader way than do financial costs and represent the full value of all resources used for an intervention, including the value (opportunity cost) of donated resources. Economic costs are important as they reflect the sustainability and replicability of interventions.

Ndyomugyenyi *et al*. 34 found that when accounting for the salaries of governmental personnel and the opportunity cost incurred by the volunteer community‐directed distributors (CDDs – Box 2), community‐directed treatment with ivermectin in Uganda cost US$0.78 per treatment (2004 prices). However, if these costs were excluded, the cost fell to just US$0.17 per treatment (2004 prices). The difference between the financial and economic costs was smaller (US$0.39 *vs*. US$0.45 (2011 prices)) in a study in Ghana 44.

A key economic cost for many MDA programmes is the value of the unpaid contribution of the volunteer CDDs. Turner *et al*. 52 found that the average economic costs relating to the volunteer CDDs unpaid time can be significant, varying between US$0.05–0.16 per treatment (cost year variable). They estimated that the time donated by volunteer CDDs to APOC (1997–2015) would be valued between US$60–90 million 52.

#### Economic value of ivermectin

Some of the identified studies considered the economic value of the donated ivermectin within their cost‐effectiveness analysis. Most used the quoted commercial value from the Mectizan Donation Program, i.e. US$1.50 (and US$0.0018 in shipping costs) per tablet. Assuming that, on average, a treatment requires 2.8 tablets, this results in an estimated average economic value of donated ivermectin of US$4.21 per treatment 30. Kim *et al*. 53 assumed a different economic value of US$1.5054 per treatment. Over the past 30 years, Merck & Co. has donated over 2.7 billion treatments of ivermectin for onchocerciasis and lymphatic filariasis 16. Based on these different assumed valuations of ivermectin this donation would be valued between US$4–11 billion.

In the context of these economic evaluations, it should be noted that it is difficult to estimate the true economic value of a donated drug 54. This is because the manufacturing costs of drugs are proprietary information, and the donating company may potentially mitigate some of the cost through charitable tax write‐offs as well as intangible benefits, such as enhanced public image among shareholders and employees 55. It is also argued that if ivermectin were not donated, it could potentially be procured from other sources at less than the proprietary cost. For example, Hernando *et al*. 55 estimate the price of an annual 9 mg ivermectin treatment with generic drugs on the global market 56 to be approximately US$0.78. Based on this, they estimated the direct cost for the tablets donated during 2005–2011 to be US$600 million, compared with the stated value of US$3.8 billion 55, and with potential tax write‐offs their net cost could be around US$180 million 55.

These arguments need to be interpreted with caution. Firstly, there is the issue of drug quality, which is a major challenge in low‐income countries, and especially with anthelmintics and similar popular drugs which commonly attract counterfeit manufacturers. Secondly, the donations themselves have a chilling effect on the markets, driving generic prices downwards. There is in fact, no established way to estimate the ‘real’ market price of these drugs, and it is notable that even the lowest estimates of the value of the donations are very substantial, measured in hundreds of millions of dollars per year. What is clear is that the ivermectin donation was the first substantial donation of its type, and its success led to the concept of the NTDs as solvable diseases of poverty, and to the massive donation of some 1.5 billion treatments for a range of NTDs by some 14 other pharmaceutical companies during the London Declaration 57.

The foundation of onchocerciasis control programmes is based on the commitment of ivermectin donation from Merck & Co. for as long as needed 17, 57. Therefore, it is debatable when the value of ivermectin should be included within an economic evaluation.

### Estimated economic benefits and cost‐benefit analyses of onchocerciasis interventions

We identified several cost‐benefit analyses of onchocerciasis interventions (Table 1). The estimated internal rate of return (IRR ‐ Box 2) ranged from between 11–20% for the OCP and 17–24% for APOC (for comparison, an IRR above 10% is considered by the World Bank as the standard for a successful public health programme 58) (Table 1). The estimated net present values (NPV) were between US$8 million to US$3.7 billion for OCP and US$54–307 million for APOC (cost years variable). However, there was variation between the different studies regarding the time frame considered and the discount rate used (Box 3), making it difficult to compare the results directly. None of the cost‐benefit analyses identified included the economic value of the donated ivermectin tablets in their base case analysis (Table 1). Waters *et al*. 18 highlighted that this value could outweigh the estimated economic benefits from both the OCP and APOC programmes.

More recently, Redekop *et al*. 59 and Kim *et al*. 60 quantified the economic benefits associated with onchocerciasis elimination. Both those studies found that eliminating onchocerciasis would generate billions in economic benefits (Tables 1 and 3).

**Table 3:** Summary of the estimated economic benefits of onchocerciasis interventions relating to productivity gains and land gains

Source	Setting and time period of the intervention	Time horizon for the benefits	Cost year	Value of the productivity gains^b^	Value of land gains
Benton & Skinner 67	OCP (1974–2004)	1974–2023	1985 US$	US$91 million (10% discount rate) to US$338 million (5% discount rate). Assumed that blindness results in complete loss of productivity. The productivity gains were valued at a subsistence wage of US$150 per year.	US$57 million (10% discount rate) to US$205 million (5% discount rate). Assumed 15 million hectares of new land made available. Assumed that new land would be settled at a rate of 3% per year, beginning five years after the programme started in the relevant area.
Kim & Benton 58	OCP (1974–2002)	1974–2012	1987 US$	Values not stated. Assumed that each case of blindness averted results in 20 years of productive life gained. Assumed 85% labour force participation^c^. The productivity gains were valued based on the ‘Agriculture value‐added factor cost’ statistic.	Values not stated (but land related benefits accounted for the majority of the study's estimated economic benefits). Assumed 25 million hectares of new land made available. Assumed 85% agricultural output^c^ and that new land utilisation would follow an S‐Curve pattern beginning after eight years of OCP intervention.
McFarland & Murray 124, a	OCP (10‐year project time period)	1974–2023	Not available	US$75 million annually (unclear if discounted). The productivity gains were valued assuming annual wages of US$150.	US$205 million (5% discount rate). Details not available.
Benton 125	APOC (1996–2007)	1996–2017	1996 US$	Values not stated. The productivity gains for each case of blindness prevented were valued at US$150 (unclear if this is the total per case or per productive year).	‐
Haddix 69, a	APOC (1996–2007)	1996–2017	1996 US$	Values not available. Details not available.	Values not available. Measured as the increase in agricultural output made available by increased productive labour. Details not available.
Kim *et al*. 60, d ^,^ e	Potential benefits of achieving elimination scenarios in Africa	2013–2045	2013 US$	Compared with the control scenario, the Elim I and II scenarios^e^ would generate US$5.9 (2.5–7.2) billion and US$6.4 (2.8–8.0) billion in productivity gains respectively (3% discount rate). The productivity gains were valued based on the GDP per capita and were adjusted for employment rates.	‐
Redekop *et al*. 59	Potential economic benefits of achieving the WHO 2020 targets	2011–2030	2005 US$	US$3.3 (2.4–5.11) billion (3% discount rate). 22% due to averted skin disease and 78% from averted visual morbidity). The productivity gains were valued based on the GDP per capita of the lowest income quintile^c^.	‐

APOC, African Programme for Onchocerciasis Control; GDP, gross domestic product; OCP, Onchocerciasis Control Programme in West Africa.

a Information for this study was taken from Waters *et al*. 18.

b Further detail regarding how the productivity gains were calculated are provided in Table 5.

c Assumption varied in the sensitivity analysis.

d Also quantified the savings to the health systems and households (out‐of‐pocket payments) resulting from decreased usage of outpatient health services (Elim I: US$60.6 (30–80.7) million, Elim II: US$64.6 (31.8–86.4) million ‐ compared with the control scenario).

e The Control, Elim I and Elim II scenarios are described in Kim *et al*. 99.

The identified studies quantified three different types of economic benefits of onchocerciasis interventions (namely, productivity gains, land use gains and reductions in outpatient services and out‐of‐pocket expenditures), but there was variation across the studies regarding how and which economic benefits were quantified (Table 3). Many of the broader benefits of onchocerciasis in terms of the Millennium Development Goals are highlighted by Dunn *et al*. 61.

#### Productivity gains

Onchocerciasis‐associated morbidity can affect an individual's economic productivity. Lenk *et al*. 62 recently conducted a systematic review of the productivity losses related to the NTDs eligible for preventive chemotherapy and a summary of the studies they identified for onchocerciasis is presented in Table 4. Unsurprisingly, the degree of the productivity loss was dependent on the type of onchocerciasis morbidity. Productivity losses were highest for blindness and lowest for skin disease.

**Table 4:** Description of studies investigating the productivity losses associated with onchocerciasis‐associated morbidity (adapted from 62)

Study	Country	Year	Study design	Population	Sample size	Sequela	Definition of productivity loss	Results
Evans 128	Guinea	1995	Observational (survey)	Household members in a highly endemic area.	319	a) Visual impairment	Self‐reported ‘inactive’ occupational status.	a) 38%
						b) Blindness		b) 79%
Kim *et al*. 129	Ethiopia	1997	Case‐control	Coffee plantation workers.	235	a) OSD (intermediate)	a) Daily wages (individuals infected with OSD (intermediate) *vs*. those without).	a) 10%
						b) OSD (severe)	b) Daily wages (individuals infected with OSD (severe) *vs*. those without).	b) 15%
Okeibunor *et al*. 130	Cameroon, DRC, Nigeria, Uganda	2011	Observational (cross‐sectional)	Primarily residents from villages where ivermectin distribution was ongoing.	1600	General onchocerciasis	a) Increase in productivity from ivermectin treatment.	a) 76%
							b) Percentage of respondents that referred ability to work better after ivermectin treatment.	b) 75.6%
Oladepo *et al*. 131	Nigeria	1993	Case‐control	Male farmers.	102	OSD	Farm size that a man can keep satisfactorily weeded (workers with *vs*. without OSD).	9117 *vs*. 13 850 m^2^ (34% loss)
Thomson 132	Cameroon	1971	Case‐control	Estate workers in an onchocerciasis endemic area.	420	Unspecified (general)	Working days (workers with *vs*. without onchocerciasis).	20%
Wogu & Okaka 133	Nigeria	2008	Observational (survey)	Rural farming community in a mesoendemic area.	200	a) OSD (itching)	a) Percentage of respondents that reported a reduction in strength and concentration at work.	a) 13.5%
						b) OSD (nodules)	b) Percentage of respondents that reported a decline in sales in business/trading.	b) 11%
						c) Visual impairment (ocular lesions)	c) Percentage of respondents that reported giving up jobs (Productivity loss not specified).	c) 14%
Workneh *et al*. 134	Ethiopia	1993	Case‐control	Male permanent coffee plantation workers.	196	OSD	Absenteeism/sick leave and net monthly pay (workers with *vs*. without OSD).	25%
WHO & World Bank 70	Nigeria, Ethiopia, Sudan	1997	Case‐control	Households in hyperendemic communities.	824	OSD	Time spent on productive activities (individuals with *vs*. without OSD signs and symptoms).	Not significant

DRC, Democratic Republic of the Congo; OSD, onchocerciasis‐associated skin disease.

A key component of estimating the economic benefits of onchocerciasis interventions is the productivity gains that result from preventing onchocerciasis morbidity. The cost‐benefit analyses identified appeared to estimate only the productivity gains associated with preventing onchocerciasis‐associated blindness. However, the productivity losses associated with onchocerciasis‐associated skin disease and visual impairment are also significant (Tables 4 and 5). Consequently, only quantifying the productivity gains associated with prevented cases of blindness underestimates the economic benefits of onchocerciasis interventions 63.

**Table 5:** Summary of the assumed productivity losses and disability weights used for onchocerciasis‐associated morbidity

Study	Low vision	Blindness	Skin disease/troublesome itch	Source
Assumed productivity loss				
Benton & Skinner 67	‐	100%	‐	
Kim & Benton 58	‐	100%	‐	
McFarland & Murray 124	Not available	Not available	Not available	
Benton 125	‐	Unclear	‐	
Haddix 69	‐	Not available	‐	
Kim *et al*. 60	Patient: 38% Caregiver: 5%	Patient: 79% Caregiver: 10%	Severe itching: 19%	128, 129, 134, 135, 136, 137
Redekop *et al*. 59	38%	79%	Moderate: 10% Mild: 0%	128, 129
Healthy life year weights^a^				
Prescott *et al*. 126, 127	‐	1.0	‐	
Evans *et al*. 71	‐	0.5	‐	
Benton 125	‐	1.0	‐	
DALY weights^a^				
GBD 1990	0.245	0.488 (treated) 0.600 (untreated)	0.068	GBD 1990 138
GBD 2000 138	0.224 (treated) 0.282 (untreated)	0.60	0.068	GBD 2000 138
McFarland & Murray 124	Not available	Not available	Not available	
Turner *et al*. 73	0.170	0.594	0.068	GBD 2004 139
Coffeng *et al*. 30	0.282	0.594	0.068	GBD 2004 139
Coffeng *et al*. 78	0.033	0.195	0.108^b^	GBD 2010 140
de Vlas *et al*. 141	0.101^c^	0.101^c^	0.079^b^	GBD 2010 140

DALYs, disability‐adjusted life years; GBD, Global Burden of Disease Study.

a Reflect the severity of the disease sequelae with 0 representing perfect health and 1 representing death.

b Used a weight representing an overall average for skin disease across more finely disaggregated strata/severity levels.

c Used a weight representing an overall average for visual morbidity (i.e. the weight was not stratified by ‘low vision’ and ‘blindness’).

Where relevant additional studies that were not performing economic evaluations were included for comparison.

In contrast, both Kim *et al*. 60 and Redekop *et al*. 59 included the productivity gains associated with preventing onchocerciasis‐associated skin disease when quantifying the economic benefits of onchocerciasis interventions. Kim *et al*. 60 assumed that severe itching was associated with a 19% productivity loss and this made up 65% of their projected income/productivity gains. In Redekop *et al*.'s 59 study, the benefits associated with preventing skin disease represented 22% of the total projected economic benefit. This assumed that ‘moderate’ skin disease is associated with a 10% productivity loss and ‘mild’ skin disease is associated with no productivity loss (Table 5).

For these types of estimates, it is important to consider which method is used to value the productivity gains. Estimating the income of individuals affected by NTDs is challenging, as many of them are in informal employment (such as subsistence farmers). The analyses identified used a variety of different methods and sources to approximate the typical income of someone with onchocerciasis‐associated morbidity (such as the per capita gross domestic product (GDP), subsistence wage, the GDP per capita of the lowest income quintile). In some cases, the income source was not clearly stated. It is important to note that different income sources can give very different estimates of an individual's typical income, even when they relate to the same type of profession/socioeconomic status 40.

Kim *et al*. 60 also considered the potential income losses of the informal caregivers of patients with low vision and blindness (Table 5). Currently, there are very few primary data quantifying this. Ibe *et al*. 64 found that within their survey of onchocerciasis patients in Nigeria, the average productivity cost among the informal caregivers was US$3.50 per month (cost year unavailable).

In all studies productivity gains were estimated using the human capital approach, which takes the patient's perspective for valuing lost productivity. It should be noted that if the friction cost approach was used (which takes the employer's perspective, i.e. only counts as lost, the hours not worked before another employee takes over the patient's work 65), the estimated productivity gains would have been lower 66. There is continued debate within the field regarding which approach is most appropriate 65. In the context of studies for NTDs, it should be highlighted that the friction cost approach is hard to apply to populations that are predominately in informal employment. Kim *et al*. 60 and Kim & Benton 58 made adjustments for employment rates/labour force participation within their estimates.

#### Land use gains

Onchocerciasis caused many people to abandon the fertile river valleys within the countries covered by the OCP 2. It has been estimated that as a result of OCP interventions, 25 million hectares of abandoned arable land was able to be reclaimed for settlement and cultivation 58, 67 (capable of feeding 17 million people annually 67, 68). Several of the cost‐benefit analyses of the OCP included the projected economic benefits resulting from the increased agricultural output from the reclaimed land. These estimates varied between US$57–205 million (cost years variable) and were sensitive to the assumed time horizon and the discount rate (Table 3). The estimate from Kim & Benton 58 was even higher but the specific value was not stated (the NPVs (1974–2012) relating only to land use gains ranged between US$3154 million (using a 3% discount rate) and US$380 million (using a 10% discount rate)). It should be highlighted that such calculations are based on various assumptions surrounding rates of agricultural land use and repopulation of the reclaimed areas (Table 3). It is also important to consider that onchocerciasis may not have been the sole cause of depopulation of these areas 9. Because the same level of abandonment of river valleys was rarely seen in non‐OCP countries 63, the analyses of the countries covered by APOC generally did not include the economic benefits related to land‐use gains. One exception is the report by Haddix 69, which is reported to have re‐evaluated APOC interventions including the estimated economic benefit resulting from increased agricultural output (Table 3).

#### Outpatient services and out‐of‐pocket expenditure

Individuals with onchocerciasis‐associated morbidity may seek care from their local health services. Kim *et al*. 60 projected the economic benefits of onchocerciasis elimination resulting from the decreased use of outpatient health services. They estimated that between 2013 and 2045, when compared with a control scenario, the elimination of onchocerciasis in Africa would save the health systems US$35.9–38.6 million in outpatient service costs, and save the patients/households US$24.7–26.0 million in out‐of‐pocket payments (2013 prices) 60. Primary data on these costs or on how often individuals with onchocerciasis seek outpatient care or use local health services are scarce. An exception is the study by Ibe *et al*. 64, which found that the average direct cost incurred by onchocerciasis patient's per outpatient visit was US$14.00 (cost year unavailable), with the majority of this being for medications.

Interestingly, a study conducted by the World Bank and WHO reported that on average, people suffering from the manifestations of onchocerciasis‐associated skin disease were found to spend an additional US$8.10 (cost year unavailable) on health‐related expenditures over a six‐month period compared with those from the same community without these manifestations 70.

### The cost‐effectiveness analyses of onchocerciasis interventions

We identified seven studies performing cost‐effectiveness analyses of onchocerciasis interventions. The majority of the studies evaluated annual MDA (Table 2). As with the cost‐benefit analyses, variation in the time frames and discount rates used (Box 3) make it difficult to compare directly some of the results.

Many older studies have used ‘healthy life years averted’ as their effectiveness metric (which was based on reductions in the number of onchocerciasis‐related blindness cases). These studies generally assumed that blindness results in complete disability, and that each year lived with blindness is equal to one full healthy life year lost, i.e. they assumed a disability weight of 1, which is equivalent to death (Table 5). One exception to this was Evans *et al*. 71, who used a lower disability weight for blindness of 0.5 (based on empirical evidence that blindness does not result in complete disability and that blind people are active both socially and economically). These studies did not appear to quantify the averted burden of other types of onchocerciasis morbidity (such as skin disease) (Table 5).

More recent studies have used disability‐adjusted life years (DALYs) averted, a standardised and more comprehensive effectiveness metric. When excluding the economic value of the donated ivermectin, the estimated cost per DALY averted of annual MDA varies between US$3 and US$30 (cost year variable). In comparison the cost per DALY averted for the MDA delivered within the Global Programme to Eliminate Lymphatic Filariasis was estimated to be US$24 when using financial costs and US$64 when using economic costs including the value of the donated drugs (2014 prices) 54. The cost‐effectiveness of onchocerciasis related MDA is also very favourable compared to other interventions conducted in low‐ and middle‐income countries (a comprehensive list of cost‐effectiveness estimates for a range of health interventions in these settings is provided within Horton *et al*. 72).

**Figure 2:**
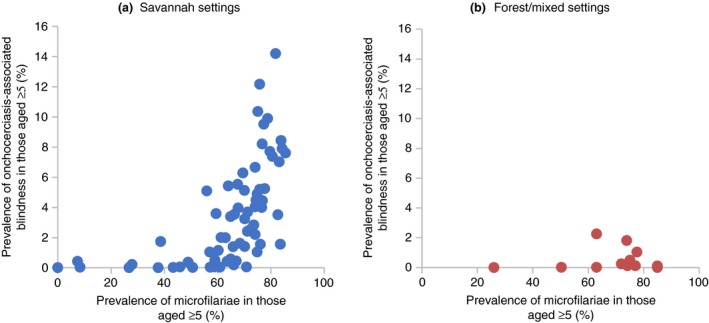
The relationship between the prevalence of onchocerciasis‐associated blindness and the prevalence of skin microfilariae in savannah (a) and forest/mixed forest‐savannah settings (b). The figures were adapted from Figures S3 and S4 in Coffeng *et al*. 30. The data were originally taken from 142, 143, 144, 145, 146, 147, 148. [Colour figure can be viewed at wileyonlinelibrary.com]

The most favourable cost‐effectiveness estimates relate to interventions in savannah settings 73. It is important to note that these estimates are not directly generalisable to onchocerciasis interventions in forest areas, where ocular pathology and morbidity is considered to be rarer (Figure 2) (but see 74). According to this assumption, intervention cost‐effectiveness in the latter areas would be lower. It should be noted that this assumption is based on limited data from forest settings (Figure 2).

The estimated cost‐effectiveness ratio was also dependent on the assumed pre‐control endemicity level, with the cost per DALY averted being lower in higher endemicity settings. In the long term, this range is narrower than might be expected as, although fewer DALYs are averted in lower endemicity settings, the total cost of the intervention is also lower (as fewer treatment rounds will be needed to achieve a similar degree of control or to reach elimination) (Table 2).

The majority of studies have taken the healthcare providers perspective, which does not consider the costs falling on those outside of the healthcare sector. All the cost‐effectiveness analyses appeared to consider only the cost of the intervention, i.e. none of them included potential savings to outpatient services, prevented out‐of‐pocket costs, or productivity gains that result from prevented morbidity. Only a few identified studies considered the economic value of the donated ivermectin. Including this significantly increases interventions estimated cost and, therefore, decreases the estimated cost‐effectiveness. For example, Turner *et al*. 54 found that the estimated cost per DALY averted increased from US$3–15 to US$29–133 (2012 prices) when including the economic value of donated ivermectin (which would still be classed as cost effective). It is also debatable when the value of ivermectin should be included within an economic evaluation, particularly under the healthcare providers perspective.

Numerous approaches have been used to quantify the effectiveness of onchocerciasis interventions. Many older studies based the effectiveness on reductions in the incidence of blindness on limited empirical data and projected similar putative reductions with continuing intervention. More recent studies have used mathematical transmission models to project the ongoing future effectiveness of interventions more accurately, explicitly modelling disease dynamics through time 75. The two main models used for this purpose (EPIONCHO and ONCHOSIM) are described in Basáñez *et al*. 76. An important advantage of this approach is the capacity to account for the indirect benefits/herd effects of interventions (the indirect benefit afforded to individuals not directly targeted by an intervention that arises from the population‐wide reduction in transmission) 75. These models can also account for longer time horizons, accounting for the continued benefits of interventions even after the control programme has stopped.

#### DALY calculations

DALYs are calculated as the sum of two components; the years of healthy life lost due to disability, and the years of life lost due to premature mortality 77. In a DALY calculation, the years of healthy life lost due to disability are calculated using standardised disability weights, ranging between 0 and 1. This reflects the severity of the different disease sequelae, with 0 representing perfect health and 1 representing death. The disability weights used for onchocerciasis DALY calculations have changed over time (Table 5).

For the 2010 GBD study, the disability weights for vision loss were notably decreased compared to previous studies, whereas the weights for skin disease were increased (Table 5). Consequently, Coffeng *et al*. 78 found that when using the newer weights, the estimated number of DALYs averted by APOC (2000–2015) increased by 9% compared to when using the GBD 2004 weights. Moreover, skin disease, instead of eye disease, became the most important contributor to the burden of onchocerciasis. Since the GBD 2010 study, onchocerciasis‐associated skin disease has not been assigned a specific single disability weight, and more general disfigurement health states (stratified by three severity levels and whether or not the disfigurement is associated with itch or pain) are used. When using these updated GBD disability weights, studies have typically estimated an overall average skin disease weight (Table 5). Subsequent GBD studies have made additional changes to the DALY calculation for onchocerciasis, particularly relating to the weights attributed to the different types of skin disease and the types of skin disease included 79. A more detailed overview of the changes to the GBD study methodology used to calculate DALYs is presented in 80, 81, 82.

Several studies have accounted for the excess mortality associated with onchocerciasis visual morbidity 3 within their DALY calculations. However, it has generally not been considered that irrespective of visual morbidity, there is an increased risk of mortality associated with increasing microfilarial load, particularly in children and young adults aged below 20 years 4, 5. Estimates of onchocerciasis burden (and the cost‐effectiveness of its control) would generally increase if this onchocerciasis‐associated excess mortality were taken into account. For example, Turner *et al*. 83 found that, depending on the pre‐control endemicity level, excess mortality accounted for 29–43% of the estimated pre‐control DALY burden of onchocerciasis. If this had not been included, the estimated cost‐effectiveness would have been reduced substantively.

#### Cost‐effectiveness thresholds

In cost‐effectiveness analyses, the cost per DALY averted is compared to a willingness to pay threshold to determine whether an intervention is cost effective. However, the most appropriate cost‐effectiveness thresholds are debated and remain somewhat arbitrary 84, 85, 86. The cost‐effectiveness threshold set by the WHO‐CHOICE 87 (a cost per DALY averted < 3 times the country's GDP per capita) is now considered to be too high 84, 85, 86, 88, 89. Most analyses within the NTD field have not used it 40, 41, 90, many opting instead for the more conservative cost‐effectiveness threshold set by the World Bank 91 (≤ US$251 per DALY averted, when adjusted for inflation to 2016 prices 92). Interestingly, recent analyses have indicated that a cost‐effectiveness threshold closer to < ½ the country's per capita GDP would be more appropriate for low‐income countries 88, 93. For comparison, the Disease Control Priorities project (Third Edition) used a threshold of US$200 per DALY averted to identify priority interventions for consideration in low‐income countries 94. Despite this ambiguity on cost‐effectiveness thresholds, onchocerciasis interventions would remain classed as cost effective by any of the proposed measures.

### Elimination and evaluation of alternative interventions

Traditional cost‐effectiveness analyses of new interventions evaluate their incremental effectiveness and incremental cost compared to the current practice (calculating incremental cost‐effectiveness ratios, Box 2). This framework has been widely and successfully used to evaluate the comparative cost‐effectiveness of new intervention strategies for disease control. However, this incremental cost‐effectiveness framework is less informative and somewhat ill‐suited for disease elimination or eradication programmes. For example, Turner *et al*. 73 found that that increasing the treatment frequency of ivermectin distribution from once to twice per year yielded very small incremental health gains (only a 3–4% increase in the number of DALYs averted) but could have a large influence on a programme's overall total cost and duration (Table 2). In such cases, where an intervention is aimed at accelerating and sustaining elimination, an incremental cost‐effectiveness ratio may not reflect its true value. Instead, as applied in Turner *et al*., the absolute cost of the intervention and the time it takes to achieve the desired elimination goal can be more informative. The same framework was applied to an economic evaluation of moxidectin 95 (Table 1), a newly registered treatment for onchocerciasis (https://www.medicinesdevelopment.com/news-180613.htm). Kastner *et al*. 96 have also highlighted that the number of DALYs averted may not fully capture the long‐term consequences and broader benefits of disease eradication programmes.

Another important aspect to consider when evaluating elimination programmes is the time horizon of the analysis 40. This is because the costs of elimination programmes are typically higher than would be needed for disease control 97. After elimination is certified, the estimated cost‐effectiveness of an elimination programme will steadily increase as the discounted benefits continue to accumulate but the costs have stopped (with the potential exception of ongoing surveillance). The benefits and potential future cost savings resulting from achieving elimination/eradication are not infinite 40, 73, as the costs/cost savings being considered must be restricted within a suitable time horizon and are typically discounted into the future (Box 3). In contrast, for disease control programmes, the intervention costs will typically be incurred for the full‐ time horizon. Because of this, elimination programmes can be cost‐saving in the long term. However, it can take time for the longer‐term benefits and cost savings associated with achieving elimination to outweigh the initial increases in costs associated with achieving elimination. Consequently, with short‐term time horizons, elimination programmes are unlikely to be more cost effective than disease control programmes.

Eradication investment cases have been developed for a number of NTDs 98, including onchocerciasis 53, 60, 99. These latter studies have compared onchocerciasis control and elimination/eradication scenarios and have quantified the duration of the programme, its financial and economic cost, the number of ivermectin treatments required, the workload of the community healthcare workers/volunteers, costs related to outpatient healthcare services, and the productivity gains resulting from preventing onchocerciasis‐associated morbidity. The overall conclusions of these studies are that eradication and elimination of onchocerciasis are both justifiable on both cost‐effectiveness and benefit‐cost analysis grounds, and that eradication tends to be more favoured over the long‐term time horizon.

### Sensitivity analysis

The majority of identified studies either did not perform a sensitivity analysis or undertook only a univariate analysis, changing one parameter at a time to evaluate its impact independent of other parameters. The main exceptions to this were Redekop *et al*. 59 and Kim *et al*. 60.

Redekop *et al*. 59 performed probabilistic sensitivity analysis (PSA) in which the values of three input parameters (the estimates of disease prevalence in 2010, the percentage of productivity loss/the amount of out‐of‐pocket payments, and the patient's income) were allowed to vary simultaneously. Kim *et al*. 60 first conducted a one‐way deterministic sensitivity analysis to examine which parameters are key drivers. They then conducted PSA to assess the robustness of the results to the joint uncertainties around all selected parameters 60.

### Limitations of this analysis

A potential source of bias of the search strategy is that it did not capture economic evaluations published outside of the searched electronic databases (i.e. grey literature such as policy documents/reports, and many non‐English language publications etc.). Efforts were made to minimise this bias by searching the bibliographies of selected studies. There could also be a degree of publication bias, with economic evaluations with negative or unfavourable results less likely to be published. It should be noted that the selection of studies was not performed independently by two researchers.

### Areas of further research

The results of the systematic review highlight that the standard onchocerciasis control strategies are consistently found to be very cost effective. However, there are some important research gaps that need further research.

#### Data on intervention costs and the economic burden of onchocerciasis

The costs of MDA delivery have been shown to vary across different settings 31, 32, 37, 41, 100. This variation potentially affects the generalisability of any cost‐effectiveness/cost‐benefit analysis 75, and future studies need to quantify the impact of this in greater detail. It should be noted that the costs of conducting MDA in areas where onchocerciasis and loiasis are co‐endemic may be higher – due to the need for enhanced surveillance and community sensitisation.

In addition, further studies are needed to investigate how integrating NTD control programmes 31, 39 may influence the costs/cost‐effectiveness of implementing different control strategies 37, 40. There is notable variation in the methodological approaches used to quantify the economic costs incurred by CDDs. It would be beneficial if future studies adopted more consistent approaches (outlined in 52).

The cost of onchocerciasis control programmes will likely increase significantly as they approach the ‘last mile’ towards elimination 37. This is partly because of the increase in the costs resulting from expanding the programmes to target harder‐to‐reach areas/groups (diseconomies of scale) 37. This is an important issue for NTD programmes in general, and further costing studies are needed to quantify it.

Further studies are needed on quantifying the medical costs incurred by those with onchocerciasis seeking treatment. Such studies could yield more robust estimates of the economic benefits of onchocerciasis control 60. It would also be beneficial if future studies sought to quantify the productivity losses incurred by informal caregivers, not just those caring for the blind. Note also that with the rise in sophistication of health systems in the endemic countries, the health system costs and the contribution of out‐of‐pocket expenses are both likely to rise 101.

Our analysis also revealed that studies used cost data collected in different years, but it was not always clear if and how costs were adjusted for inflation. Future studies should report this more explicitly 102 and within a given study the costs would be standardised to a consistent year.

#### Economic evaluations of alternative interventions

In certain epidemiological and programmatic circumstances, alternative strategies to annual MDA will be required to achieve the current goals for onchocerciasis control/elimination 21, 22, 103. Such strategies include increased frequency of MDA (up to four times per year), localised low‐cost vector control and treatment strategies using moxidectin, anti‐*Wolbachia* therapies and new macrofilaricidal drugs 104, 105, 106. However, there currently are very few costing studies and economic evaluations relating to these alternative interventions 32, 107. Studies are needed to evaluate the cost and cost‐effectiveness of such strategies. When such studies are performed, it will be vital that the generalisability of the estimated cost across different programmatic settings is considered 37. It will also be important to consider the value of these alternative interventions not only in reducing the disease burden where they are implemented, but also in their capacity to help eliminate onchocerciasis more quickly. This will be particularly important in reducing the risk that ‘hot spots’ of sustained transmission seed and re‐establish transmission in areas where onchocerciasis has been eliminated.

This area of research is particularly important for interventions targeting onchocerciasis in *Loa loa* co‐endemic areas. It has been recently predicted that, at the 2025 horizon, two‐thirds of onchocerciasis remaining cases will be living in hypoendemic areas for onchocerciasis where the risk of *Loa*‐related post‐ivermectin severe adverse events (SAEs) has so far been considered to outweigh the benefits for the communities. The paradigm shift from control to elimination implies that hypoendemic areas start receiving community treatment with ivermectin. A test‐and‐not‐treat strategy for onchocerciasis has recently been successfully piloted in a health area of Central Cameroon where community‐directed treatment with ivermectin had to be permanently halted after a series of SAEs occurred in 1999 108. Costing studies of this approach are ongoing. Critical questions when addressing the cost‐effectiveness of this intervention include the counterfactual – what it would cost to leave all those populations untreated? – and cost‐effectiveness relative to other locally important health issues.

Future economic analyses of alternative interventions need to be tailored to the key policy questions from the different decision makers and stakeholders. These different questions may require different methodological approaches and perspectives for the analyses. This further emphasises the need for transparent reporting of methodology in economic evaluations.

#### Quantifying the health benefits of interventions

Many older studies only quantified the health and economic benefits resulting from the number of blindness cases averted. More advanced disease models have been developed that account for averted visual impairment, skin disease, and excess human mortality. However, further refinements are needed to better capture the relationship between infection and skin disease 109, and to account for related neurological disorders such as epilepsy and nodding syndrome 6, 7, 8.

When evaluating an intervention aimed at reducing morbidity, the number of DALYs averted is often the best effectiveness metric, as it allows cost‐effectiveness estimates to be directly compared to estimates relating to other interventions/diseases as well as to standardised cost‐effectiveness thresholds. This makes the results of economic evaluations easier to interpret by policymakers. However, DALYs do have limitations and there are controversies surrounding their calculation 110. For example:

- The universal disability weights do not account for how the local context may influence the burden of a disease. Consequently, the potential that the burden of a disease or its sequelae may be worse for those that are living in poverty is not accounted for. It has been argued that this aspect of DALY calculations may significantly underestimate the burden of poverty‐related diseases 110, 111.
- The DALY disability weights do not fully account for the psycho‐social implications of a disease or its sequelae 112 and its overall impact on quality of life. They also do not explicitly account for the impact of the disease on patients’ informal caregivers. In particular, the updated disability weight for blindness (decreasing from 0.60 to 0.19) has been controversial within the field 113, 114. A possible reason for this significant change is that within the updated GBD framework (post‐GBD 2010), the disability weights are intended to be solely measures of losses of ‘optimal health’ and are not intended to represent losses of well‐being/welfare 80, 115.

Interestingly, the same age group (those aged below 20 years) for which there is a statistically significant higher risk of mortality for a given microfilarial load in comparison to those aged 20 years and older 5, is the group with the onset and higher incidence of nodding syndrome, a type of epilepsy that is increasingly recognised as associated with onchocerciasis 116, 117. Preliminary studies of the disease burden of onchocerciasis‐associated epilepsy have been conducted 118. These studies should be followed by economic evaluations of both onchocerciasis‐associated epilepsy and the impact of onchocerciasis interventions.

#### Joint/auxiliary benefits

Ivermectin is a broad‐spectrum antiparasitic drug that also has an impact on other co‐endemic parasitic infections (such as soil‐transmitted helminthiases, lymphatic filariasis, loiasis and scabies). Krotneva *et al*. 119 assessed the auxiliary benefits of APOC and estimated that between 1995 and 2010, ivermectin mass treatment (in APOC regions) cumulatively averted approximately 500 000 DALYs from co‐endemic soil‐transmitted helminth infections, lymphatic filariasis, and scabies. This highlights that the overall cost‐effectiveness of onchocerciasis interventions may be even higher than previously reported. Further quantification of these auxiliary benefits would be useful to improve estimations of the impact of onchocerciasis interventions.

#### Modelling and elimination thresholds

Dynamic transmission models have an important role, particularly for the evaluation of novel interventions and how they compare to the standard strategy of MDA with ivermectin. These models (reviewed in Basáñez *et al*. 76) have undergone extensive refinement in recent years to better capture parasite population dynamics during interventions 120 and to produce more robust projections on the likelihood of elimination. These modelling efforts will be particularly useful in identifying epidemiological and programmatic circumstances in which alternative strategies will be required to reach elimination (for example in highly endemic settings, settings with suboptimal responses to ivermectin 121, 122, or where the initiation of programmes has previously been delayed 103). Moreover, transmission models (as opposed to so‐called ‘static’ models) capture explicitly our current understanding of the changing parasite dynamics during interventions 75.

## Conclusions

The cost benefit and cost effectiveness of onchocerciasis interventions have consistently been found to be very favourable. This finding provides strong evidential support for the ongoing efforts to eliminate onchocerciasis from endemic areas.

MDA against other NTDs has also been found to be cost effective 40, 54, 90 and, therefore, a logical next step would be to quantify the net cost‐effectiveness of more closely integrating these programmes, which are already often running side‐by‐side in co‐endemic areas. Indeed, the primary rationale for the aggregation of common infectious diseases of the poor under the denomination of ‘Neglected Tropical Diseases’ was the perception that treating many diseases using a single common delivery system would be inherently cost effective. It would be important to future policy making to explore the evidence base for that perception. Further systematic reviews of this type on other NTDs would also be useful.

We identify three main research gaps in this area. First, the need to be more inclusive in quantifying burden. Originally, studies only quantified the benefits of preventing blindness, and then also captured onchocerciasis‐associated skin disease. An improved understanding of other factors, such as onchocerciasis‐associated epilepsy 117, 118 would enhance the precision of the calculated benefits. Second, the evaluation of interventions targeting *Loa loa* co‐endemic areas which will become more important in the end‐game for elimination. Finally, the need to increase the comparability of economic analyses. Greater adherence to standardised guidelines for reporting the results of economic evaluations (such as CHEERS for cost‐effectiveness analysis 123) would be beneficial and increase the reliability and reproducibility of reported findings.

Programmes to eliminate onchocerciasis have always been in the vanguard of global efforts to eliminate diseases of poverty. Lessons learned here from the economic analysis of onchocerciasis programmes have direct relevance to the design of programmes addressing all the other NTDs.

## Supporting information


**Appendix S1.** PRISMA checklist.

## References

[1] Duke BO . Human onchocerciasis–an overview of the disease. Acta Leiden 1990: 59: 9–24. 2198761

[2] World Health Organization . Signs, symptoms and treatment of onchocerciasis. (Available from: http://www.who.int/onchocerciasis/symptoms/en/).

[3] Kirkwood B , Smith P , Marshall T , Prost A . Relationships between mortality, visual acuity and microfilarial load in the area of the Onchocerciasis Control Programme. Trans R Soc Trop Med Hyg 1983: 77: 862–868. 6665841 10.1016/0035-9203(83)90308-5

[4] Little MP , Breitling LP , Basáñez MG , Alley ES , Boatin BA . Association between microfilarial load and excess mortality in onchocerciasis: an epidemiological study. Lancet 2004: 363: 1514–1521. 15135599 10.1016/S0140-6736(04)16151-5

[5] Walker M , Little MP , Wagner KS , Soumbey‐Alley EW , Boatin BA , Basáñez MG . Density‐dependent mortality of the human host in onchocerciasis: relationships between microfilarial load and excess mortality. PLoS Negl Trop Dis 2012: 6: e1578. 22479660 10.1371/journal.pntd.0001578 PMC3313942

[6] Colebunders R , Hendy A , van Oijen M . Nodding Syndrome in Onchocerciasis Endemic Areas. Trends Parasitol 2016: 32: 581–583. 27289272 10.1016/j.pt.2016.05.013

[7] Kaiser C , Pion SDS , Boussinesq M . Case‐control studies on the relationship between onchocerciasis and epilepsy: systematic review and meta‐analysis. PLoS Negl Trop Dis 2013: 7: e2147. 23556028 10.1371/journal.pntd.0002147 PMC3610636

[8] Colebunders R , Hendy A , Mokili JL *et al* Nodding syndrome and epilepsy in onchocerciasis endemic regions: comparing preliminary observations from South Sudan and the Democratic Republic of the Congo with data from Uganda. BMC Res Notes 2016: 9: 182. 27005304 10.1186/s13104-016-1993-7 PMC4802870

[9] Basáñez M‐G , Pion SDS , Churcher TS , Breitling LP , Little MP , Boussinesq M . River blindness: a success story under threat? PLoS Med 2006: 3: e371. 17002504 10.1371/journal.pmed.0030371 PMC1576321

[10] Boatin B . The Onchocerciasis Control Programme in West Africa (OCP). Ann Trop Med Parasitol 2008: 102(Suppl 1): 13–17. 18718148 10.1179/136485908X337427

[11] Richards FO Jr , Boatin B , Sauerbrey M , Seketeli A . Control of onchocerciasis today: status and challenges. Trends Parasitol 2001: 17: 558–563. 11756018 10.1016/s1471-4922(01)02112-2

[12] Sauerbrey M . The Onchocerciasis Elimination Program for the Americas (OEPA). Ann Trop Med Parasitol 2008: 102(Suppl 1): 25–29. 18718151 10.1179/136485908X337454

[13] Roungou J‐B , Yameogo L , Mwikisa C , Boakye DA , Bundy DAP . 40 years of the APOC partnership. PLoS Negl Trop Dis 2015: 9: e0003562. 25974289 10.1371/journal.pntd.0003562 PMC4431867

[14] Lawrence J , Sodahlon YK . Onchocerciasis: the beginning of the end. Int Health 2018: 10(Suppl_1): i1–i2. 29471347 10.1093/inthealth/ihx070

[15] Colatrella B . The Mectizan Donation Program: 20 years of successful collaboration ‐ a retrospective. Ann Trop Med Parasitol 2008: 102(Suppl 1): 7–11. 18718147 10.1179/136485908X337418

[16] Gustavsen KM , Colatrella BD , McCoy T . For as long as necessary: examining 30 years of msd's focus on achieving elimination of onchocerciasis and lymphatic filariasis. Int Health 2018: 10(Suppl_1): i3–i6. 29186497 10.1093/inthealth/ihx038

[17] World Health Organization . Contribution of pharmaceutical companies to the control of neglected tropical diseases. (Available from: http://www.who.int/neglected_diseases/pharma_contribution/en/).

[18] Waters HR , Rehwinkel JA , Burnham G . Economic evaluation of Mectizan distribution. Trop Med Int Health 2004: 9: A16–A25. 10.1111/j.1365-3156.2004.01210.x 15078276

[19] Diawara L , Traoré MO , Badji A *et al* Feasibility of onchocerciasis elimination with ivermectin treatment in endemic foci in Africa: first evidence from studies in Mali and Senegal. PLoS Negl Trop Dis 2009: 3: e497. 19621091 10.1371/journal.pntd.0000497 PMC2710500

[20] Traore MO , Sarr MD , Badji A *et al* Proof‐of‐principle of onchocerciasis elimination with ivermectin treatment in endemic foci in Africa: final results of a study in Mali and Senegal. PLoS Negl Trop Dis 2012: 6: e1825. 23029586 10.1371/journal.pntd.0001825 PMC3441490

[21] African Programme for Onchocerciasis Control (APOC) . Final Communiqué, Eighteenth Session of the Joint Action Forum 11‐13 December 2012. 2012. (Available from: http://www.who.int/apoc/publications/recommendations/en/index.html).

[22] World Health Organization . Accelerating work to overcome the global impact of neglected tropical diseases– A roadmap for implementation 2012. (Available from: http://www.who.int/neglected_diseases/NTD_RoadMap_2012_Fullversion.pdf).

[23] World Health Organization . Update on the global status of implementation of preventive chemotherapy (PC). (Available from: https://www.who.int/neglected_diseases/preventive_chemotherapy/PC_Update.pdf).

[24] Hanson C , Weaver A , Zoerhoff KL *et al* Integrated implementation of programs targeting neglected tropical diseases through preventive chemotherapy: identifying best practices to roll out programs at national scale. Am J Trop Med Hyg 2012: 86: 508–513. 22403327 10.4269/ajtmh.2012.11-0589 PMC3284372

[25] Linehan M , Hanson C , Weaver A *et al* Integrated implementation of programs targeting neglected tropical diseases through preventive chemotherapy: proving the feasibility at national scale. Am J Trop Med Hyg 2011: 84: 5–14. 21212194 10.4269/ajtmh.2011.10-0411 PMC3005506

[26] World Health Organization . Expanded Special Project for Elimination of Neglected Tropical Diseases. (Available from: https://www.afro.who.int/health-topics/expanded-special-project-elimination-neglected-tropical-disease).

[27] Bangert M , Molyneux DH , Lindsay SW , Fitzpatrick C , Engels D . The cross‐cutting contribution of the end of neglected tropical diseases to the sustainable development goals. Infect Dis Poverty 2017: 6: 73. 28372566 10.1186/s40249-017-0288-0 PMC5379574

[28] Amazigo U , Noma M , Boatin BA , Etya'ale DE , Seketeli A , Dadzie KY . Delivery systems and cost recovery in Mectizan treatment for onchocerciasis. Ann Trop Med Parasitol 1998: 92(Suppl 1): S23–S31. 9861264

[29] Bundy DAP , Dhomun B , Daney X , Schultz LB , Tembon A . Investing in onchocerciasis control: financial management of the African Programme for Onchocerciasis Control (APOC). PLoS Negl Trop Dis 2015: 9: e0003508. 25974134 10.1371/journal.pntd.0003508 PMC4431882

[30] Coffeng LE , Stolk WA , Zoure HG *et al* African Programme For Onchocerciasis Control 1995‐2015: model‐estimated health impact and cost. PLoS Negl Trop Dis 2013: 7: e2032. 23383355 10.1371/journal.pntd.0002032 PMC3561133

[31] Keating J , Yukich JO , Mollenkopf S , Tediosi F . Lymphatic filariasis and onchocerciasis prevention, treatment, and control costs across diverse settings: a systematic review. Acta Trop 2014: 135: 86–95. 24699086 10.1016/j.actatropica.2014.03.017

[32] Fitzpatrick C , Madin‐Warburton M , Schneiderd T *et al* Benchmarks for the cost per person of mass treatment against neglected tropical diseases: a literature review and metaregression with web‐based software application. PLoS Negl Trop Dis 2016: 12: e0005037. 10.1371/journal.pntd.0005037 PMC5137870 27918573

[33] Fitzpatrick C , Nwankwo U , Lenk E , de Vlas SJ . An Investment Case for Ending Neglected Tropical Diseases. Disease Control Priorities (3rd edn), (Volume 6): Major Infectious Diseases. Washington, DC: World Bank, 2017; 411–431. 30212103

[34] Ndyomugyenyi R , Lakwo T , Habomugisha P , Male B . Progress towards the elimination of onchocerciasis as a public‐health problem in Uganda: opportunities, challenges and the way forward. Ann Trop Med Parasitol 2007: 101: 323–333. 17524247 10.1179/136485907X176355

[35] Katabarwa M , Mutabazi D , Richards F Jr . The community‐directed, ivermectin‐treatment programme for onchocerciasis control in Uganda–an evaluative study (1993‐1997). Ann Trop Med Parasitol 1999: 93: 727–735. 10715701 10.1080/00034989957989

[36] Katabarwa MN , Habomugisha P , Richards FO Jr . Implementing community‐directed treatment with ivermectin for the control of onchocerciasis in Uganda (1997‐2000): an evaluation. Ann Trop Med Parasitol 2002: 96: 61–73. 11998803 10.1179/000349802125000529

[37] Turner HC , Toor J , Hollingsworth TD , Anderson RM . Economic evaluations of mass drug administration: the importance of economies of scale and scope. Clin Infect Dis 2017: 66: 1298–1303. 10.1093/cid/cix1001 PMC5888956 29126255

[38] Conteh L , Engels T , Molyneux DH . Socioeconomic aspects of neglected tropical diseases. Lancet 2010: 375: 239–247. 20109925 10.1016/S0140-6736(09)61422-7

[39] Brady MA , Hooper PJ , Ottesen EA . Projected benefits from integrating NTD programs in sub‐Saharan Africa. Trends Parasitol 2006: 22: 285–291. 16730230 10.1016/j.pt.2006.05.007

[40] Gedge LM , Bettis AA , Bradley MH , Hollingsworth TD , Turner HC . Economic evaluations of lymphatic filariasis interventions: a systematic review and research needs. Parasit Vectors 2018: 11: 75. 29391042 10.1186/s13071-018-2616-z PMC5793442

[41] Turner HC , Truscott JE , Hollingsworth TD , Bettis AA , Brooker SJ , Anderson RM . Cost and cost‐effectiveness of soil‐transmitted helminth treatment programmes: systematic review and research needs. Parasit Vectors 2015: 8: 355. 26137945 10.1186/s13071-015-0885-3 PMC4499443

[42] Evans D , McFarland D , Adamani W *et al* Cost‐effectiveness of triple drug administration (TDA) with praziquantel, ivermectin and albendazole for the prevention of neglected tropical diseases in Nigeria. Ann Trop Med Parasitol 2011: 105: 537–547. 22325813 10.1179/2047773211Y.0000000010 PMC4089800

[43] Bundy DAP , Appleby LJ , Bradley M *et al* Mass Deworming Programs in Middle Childhood and Adolescence. Chapter 13 in Disease Control Priorities, 3rd edn (Volume 8): Child and Adolescent Health and Development. Washington, DC: World Bank, 2017; 165–182. 30212135

[44] Turner HC , Osei‐Atweneboana MY , Walker M *et al* The cost of annual versus biannual community‐directed treatment of onchocerciasis with ivermectin: Ghana as a case study. PLoS Negl Trop Dis 2013: 7: e2452. 24069497 10.1371/journal.pntd.0002452 PMC3777881

[45] Godin C . Cameroon and Chad: cost recovery. Ann Trop Med Parasitol 1998: 92(Suppl 1): S163–S164. 9861286

[46] Hopkins AD . Mectizan delivery systems and cost recovery in the Central African Republic. Ann Trop Med Parasitol 1998: 92(Suppl 1): S97–S100. 9861274 10.1080/00034989859627

[47] Onwujekwe OE , Shu EN , Okonkwo PO . Community financing of local ivermectin distribution in Nigeria: potential payment and cost‐recovery outlook. Trop Doct 2000: 30: 91–94. 10842555 10.1177/004947550003000212

[48] Onwujekwe OE , Shu EN , Nwagbo D , Akpala CO , Okonkwo PO . Willingness to pay for community‐based ivermectin distribution: a study of three onchocerciasis‐endemic communities in Nigeria. Trop Med Int Health 1998: 3: 802–808. 9809913 10.1046/j.1365-3156.1998.00304.x

[49] Onwujekwe OE , Shu EN , Okonkwo PO . Willingness to pay for the maintenance of equity in a local ivermectin distribution scheme in Toro, Northern Nigeria. Public Health 1999: 113: 193–194. 10483083

[50] WHO‐CHOICE . Making choices in health: WHO guide to cost‐effectiveness analysis. Geneva: World Health Organization, 2003. (Available from: https://www.who.int/choice/publications/p_2003_generalised_cea.pdf).

[51] Drummond MF , Sculpher MJ , Torrance GW , O'Brien BJ , Stoddart GL . Methods for the Economic Evaluation of Health Care Programme, 3rd edn Oxford: Oxford University Press, 2005.

[52] Turner HC , Toor J , Bettis AA *et al* Valuing the unpaid contribution of community health volunteers to mass drug administration programs. Clin Infect Dis 2018; [Epub ahead of print]. 10.1093/cid/ciy741 PMC6481994 30169566

[53] Kim YE , Sicuri E , Tediosi F . Financial and Economic Costs of the Elimination and Eradication of Onchocerciasis (River Blindness) in Africa. PLoS Negl Trop Dis 2015: 9: e0004056. 26360917 10.1371/journal.pntd.0004056 PMC4567329

[54] Turner HC , Bettis AA , Chu BK *et al* Investment success in public health: an analysis of the cost‐effectiveness and cost‐benefit of the Global Programme to Eliminate Lymphatic Filariasis. Clin Infect Dis 2017: 64: 728–735. 27956460 10.1093/cid/ciw835 PMC5404931

[55] Hernando Y , Colwell K , Wright BD . Doing well while fighting river blindness: the alignment of a corporate drug donation programme with responsibilities to shareholders. Trop Med Int Health 2016: 21: 1304–1310. 27458720 10.1111/tmi.12759

[56] Management Sciences for Health . The International Medical Products Price Guide. (Available from: http://mshpriceguide.org/en/home/).

[57] Uniting to Combat NTDs . London Declaration on Neglected Tropical Diseases. (Available from: https://unitingtocombatntds.org/london-declaration-neglected-tropical-diseases/).

[58] Kim A , Benton B . Cost‐benefit analysis of the onchocerciasis control program (OCP). The World Bank; 1995. (Available from: http://documents.worldbank.org/curated/en/205811468739219136/pdf/multi0page.pdf).

[59] Redekop WK , Lenk EJ , Luyendijk M *et al* The Socioeconomic Benefit to Individuals of Achieving the 2020 Targets for Five Preventive Chemotherapy Neglected Tropical Diseases. PLoS Negl Trop Dis 2017: 11: e0005289. 28103243 10.1371/journal.pntd.0005289 PMC5313231

[60] Kim YE , Stolk WA , Tanner M , Tediosi F . Modelling the health and economic impacts of the elimination of river blindness (onchocerciasis) in Africa. BMJ Glob Health 2017: 2: e000158. 10.1136/bmjgh-2016-000158 PMC5435253 28589011

[61] Dunn C , Callahan K , Katabarwa M *et al* The Contributions of Onchocerciasis Control and Elimination Programs toward the Achievement of the Millennium Development Goals. PLoS Negl Trop Dis 2015: 9: e0003703. 25996946 10.1371/journal.pntd.0003703 PMC4440802

[62] Lenk EJ , Redekop WK , Luyendijk M , Rijnsburger AJ , Severens JL . Productivity loss related to neglected tropical diseases eligible for preventive chemotherapy: a systematic literature review. PLoS Negl Trop Dis 2016: 10: e0004397. 26890487 10.1371/journal.pntd.0004397 PMC4758606

[63] Remme JHF , Feenstra P , Lever PR , Medici AC , Morel CM . Tropical diseases targeted for elimination: chagas disease, lymphatic dilariasis, onchocerciasis, and leprosy In: Dean T Jamison, Joel G Breman, Anthony R Measham, George Alleyne, Mariam Claeson, David B Evans, Prabhat Jha, Anne Mills, Philip Musgrove (eds). Disease Control Priorities in Developing Countries, 2nd edn New York: Oxford University Press, 2006; 433–449. 21250324

[64] Ibe O , Onwujekwe O , Uzochukwu B , Ajuba M , Okonkwo P . Exploring Consumer Perceptions and Economic Burden of Onchocerciasis on Households in Enugu State, South‐East Nigeria. PLoS Negl Trop Dis 2015: 9: e0004231. 26618633 10.1371/journal.pntd.0004231 PMC4664248

[65] Krol M , Brouwer W , Rutten F . Productivity costs in economic evaluations: past, present, future. Pharmacoeconomics 2013: 31: 537–549. 23620213 10.1007/s40273-013-0056-3

[66] van den Hout WB . The value of productivity: human‐capital versus friction‐cost method. Ann Rheum Dis 2010: 69(Suppl 1): i89–i91. 19995754 10.1136/ard.2009.117150

[67] Benton B , Skinner ED . Cost‐benefits of onchocerciasis control. Acta Leiden 1990: 59: 405–411. 2116059

[68] World Health Organization . Prevention, control and elimination of onchocerciasis. (Available from: http://www.who.int/onchocerciasis/control/en/).

[69] Haddix A . Economic Re‐evaluation of the African Programme for Onchocerciasis Control. School of Public Health of Emory University: Rollins Atlanta, 1997.

[70] World Health Organization, World Bank . Economic Impact of Onchocercal Skin Disease (OSD). Report of a Multi‐country Study. TDR Applied Field Research Report World Health Organization, Geneva. 1997.

[71] Evans TG , Murray CJL . A critical re‐examination of the economics of blindness prevention under the onchocerciasis control programme. Soc Sci Med 1987: 25: 241–249. 3114891 10.1016/0277-9536(87)90227-9

[72] Horton S , Gelband H , Jamison D , Levin C , Nugent R , Watkins D . Ranking 93 health interventions for low‐ and middle‐income countries by cost‐effectiveness. PLoS ONE 2017: 12: e0182951. 28797115 10.1371/journal.pone.0182951 PMC5552255

[73] Turner HC , Walker M , Churcher TS *et al* Reaching the London declaration on neglected tropical diseases goals for onchocerciasis: an economic evaluation of increasing the frequency of Ivermectin treatment in Africa. Clin Infect Dis 2014: 59: 923–932. 24944228 10.1093/cid/ciu467 PMC4166981

[74] Cheke RA , Garms R . Indices of onchocerciasis transmission by different members of the Simulium damnosum complex conflict with the paradigm of forest and savanna parasite strains. Acta Trop 2013: 125: 43–52. 22995985 10.1016/j.actatropica.2012.09.002

[75] Turner HC , Walker M , French MD , Blake IM , Churcher TS , Basáñez MG . Neglected tools for neglected diseases: mathematical models in economic evaluations. Trends Parasitol 2014: 30: 562–570. 25455565 10.1016/j.pt.2014.10.001

[76] Basáñez MG , Walker M , Turner HC , Coffeng LE , de Vlas SJ , Stolk WA . River blindness: mathematical models for control and elimination. Adv Parasitol 2016: 94: 247–341. 27756456 10.1016/bs.apar.2016.08.003

[77] Gold MR , Stevenson D , Fryback DG . HALYS and QALYS and DALYS, Oh My: similarities and differences in summary measures of population Health. Annu Rev Public Health 2002: 23: 115–134. 11910057 10.1146/annurev.publhealth.23.100901.140513

[78] Coffeng LE , Stolk WA , Zoure HG *et al* African programme for onchocerciasis control 1995‐2015: updated health impact estimates based on new disability weights. PLoS Negl Trop Dis 2014: 8: e2759. 24901642 10.1371/journal.pntd.0002759 PMC4046979

[79] Global Burden of Disease Study 2013 Collaborators . Global, regional, and national incidence, prevalence, and years lived with disability for 301 acute and chronic diseases and injuries in 188 countries, 1990‐2013: a systematic analysis for the Global Burden of Disease Study 2013. Lancet 2015: 386: 743–800. 26063472 10.1016/S0140-6736(15)60692-4 PMC4561509

[80] World Health Organization . WHO methods and data sources for global burden of disease estimates 2000‐2011. 2013. (Available from: https://www.who.int/healthinfo/statistics/GlobalDALYmethods_2000_2011.pdf).

[81] Voigt K , King NB . Disability weights in the global burden of disease 2010 study: two steps forward, one step back? Bull World Health Organ 2014: 92: 226–228. 24700983 10.2471/BLT.13.126227 PMC3949595

[82] Murray CJ , Lopez AD . Measuring global health: motivation and evolution of the Global Burden of Disease Study. Lancet 2017: 390: 1460–1464. 28919120 10.1016/S0140-6736(17)32367-X

[83] Turner HC , Walker M , Churcher TS , Basáñez M‐G . Modelling the impact of ivermectin on river blindness and its burden of morbidity and mortality in African Savannah: EpiOncho projections. Parasit Vectors 2014: 7: 241. 24886747 10.1186/1756-3305-7-241 PMC4037555

[84] Newall AT , Jit M , Hutubessy R . Are current cost‐effectiveness thresholds for low‐ and middle‐income countries useful? Examples from the world of vaccines. Pharmacoeconomics 2014: 32: 525–531. 24791735 10.1007/s40273-014-0162-x

[85] Marseille E , Larson B , Kazi DS , Kahn JG , Rosen S . Thresholds for the cost–effectiveness of interventions: alternative approaches. Bull World Health Organ 2015: 93: 118–124. 25883405 10.2471/BLT.14.138206 PMC4339959

[86] Leech AA , Kim DD , Cohen JT , Neumann PJ . Use and Misuse of Cost‐Effectiveness Analysis Thresholds in Low‐ and Middle‐Income Countries: Trends in Cost‐per‐DALY Studies. Value in Health. 2018. 10.1016/j.jval.2017.12.016 PMC6041503 30005746

[87] Hutubessy R , Chisholm D , Edejer TT‐T , Who C . Generalized cost‐effectiveness analysis for national‐level priority‐setting in the health sector. Cost Eff Resour Alloc 2003: 1: 8. 14687420 10.1186/1478-7547-1-8 PMC320499

[88] Woods B , Revill P , Sculpher M , Claxton K . Country‐level cost‐effectiveness thresholds: initial estimates and the need for further research. Value Health 2016: 19: 929–935. 27987642 10.1016/j.jval.2016.02.017 PMC5193154

[89] Shillcutt SD , Walker DG , Goodman CA , Mills AJ . Cost‐effectiveness in low‐ and middle‐income countries: a review of the debates surrounding decision rules. Pharmacoeconomics 2009: 27: 903–917. 19888791 10.2165/10899580-000000000-00000 PMC2810517

[90] Fitzpatrick C , Nwankwo U , Lenk EJ , de Vlas SJ , Bundy DAP . An Investment Case for Ending Neglected Tropical Diseases. Disease Control Priorities, 3rd edn (Volume 6): World Bank: Washington, DC, 2017. 30212103

[91] World Bank . World development report 1993: investing in health. New York: Oxford University Press; 1993. (Available from: http://documents.worldbank.org/curated/en/468831468340807129/World-development-report-1993-investing-in-health)

[92] Holmstrom O . Point‐of‐care mobile digital microscopy and deep learning for the detection of soil‐transmitted helminths and Schistosoma haematobium. PLoS Negl Trop Dis 2017: 10: 1337325. 10.1080/16549716.2017.1337325 PMC5645671 28838305

[93] Ochalek J , Lomas J , Claxton K . Cost per DALY averted thresholds for low‐ and middle‐income countries: evidence from cross country data. University of York, Centre for Health Economics, Working Paper 122 2015(Available from: https://pure.york.ac.uk/portal/en/publications/cost-per-daly-averted-thresholds-for-low-and-middleincome-countries(12487fa5-e63f-4ac3-9fa4-03b2795065eb).html).

[94] Horton S . Cost‐Effectiveness Analysis in Disease Control Priorities, 3rd edn, Volume 9, Disease Control Priorities. Washington, DC: World Bank, 2017. 30212156

[95] Turner HC , Walker M , Attah SK *et al* The potential impact of moxidectin on onchocerciasis elimination in Africa: an economic evaluation based on the Phase II clinical trial data. Parasit Vectors 2015: 8: 167. 25889256 10.1186/s13071-015-0779-4 PMC4381491

[96] Kastner RJ , Stone CM , Steinmann P , Tanner M , Tediosi F . Chapter Seven ‐ lessons learned from developing an eradication investment case for lymphatic filariasis In: Maria Gloria Basáñez, Roy M. Anderson (eds) Advances in Parasitology, Volume 94 London UK: Academic Press, 2016; 393–417. 27756458 10.1016/bs.apar.2016.08.004

[97] Sicuri E , Evans DB , Tediosi F . Can Economic Analysis Contribute to Disease Elimination and Eradication? A Systematic Review. PLoS ONE 2015: 10: e0130603. 26070135 10.1371/journal.pone.0130603 PMC4466479

[98] Tediosi F , Steinmann P , de Savigny D , Tanner M . Developing eradication investment cases for onchocerciasis, lymphatic filariasis, and human African trypanosomiasis: rationale and main challenges. PLoS Negl Trop Dis 2013: 7: e2446. 24244762 10.1371/journal.pntd.0002446 PMC3820723

[99] Kim YE , Remme JH , Steinmann P , Stolk WA , Roungou JB , Tediosi F . Control, elimination, and eradication of river blindness: scenarios, timelines, and ivermectin treatment needs in Africa. PLoS Negl Trop Dis 2015: 9: e0003664. 25860569 10.1371/journal.pntd.0003664 PMC4393239

[100] Goldman AS , Guisinger VH , Aikins M *et al* National mass drug administration costs for lymphatic filariasis elimination. PLoS Negl Trop Dis 2007: 1: e67. 17989784 10.1371/journal.pntd.0000067 PMC2041814

[101] Global Burden of Disease Health Financing Collaborator Network . Evolution and patterns of global health financing 1995‐2014: development assistance for health, and government, prepaid private, and out‐of‐pocket health spending in 184 countries. Lancet 2017: 389: 1981–2004. 28433256 10.1016/S0140-6736(17)30874-7 PMC5440770

[102] Kumaranayake L . The real and the nominal? Making inflationary adjustments to cost and other economic data. Health Policy Plan 2000: 15: 230–234. 10837047 10.1093/heapol/15.2.230

[103] Verver S , Walker M , Kim YE *et al* How can onchocerciasis elimination in Africa be accelerated? Modeling the impact of increased ivermectin treatment frequency and complementary vector control. Clin Infect Dis 2018: 66(suppl_4): S267–S274. 29860291 10.1093/cid/cix1137 PMC5982715

[104] Boussinesq M , Fobi G , Kuesel AC . Alternative treatment strategies to accelerate the elimination of onchocerciasis. Int Health 2018: 10(suppl_1): i40–i48. 29471342 10.1093/inthealth/ihx054 PMC5881258

[105] Kuesel AC . Research for new drugs for elimination of onchocerciasis in Africa. Int J Parasitol Drugs Drug Resist 2016: 6: 272–286. 27693536 10.1016/j.ijpddr.2016.04.002 PMC5196484

[106] Jacob BG , Loum D , Lakwo TL *et al* Community‐directed vector control to supplement mass drug distribution for onchocerciasis elimination in the Madi mid‐North focus of Northern Uganda. PLoS Negl Trop Dis 2018: 12: e0006702. 30148838 10.1371/journal.pntd.0006702 PMC6128654

[107] Turner HC , Osei‐Atweneboana MY , Walker M *et al* The cost of annual versus biannual community‐directed treatment with ivermectin: Ghana as a case study. PLoS Negl Trop Dis 2013: 7: e2452. 24069497 10.1371/journal.pntd.0002452 PMC3777881

[108] Kamgno J , Pion SD , Chesnais CB *et al* A Test‐and‐Not‐Treat Strategy for Onchocerciasis in Loa loa‐Endemic Areas. N Engl J Med 2017: 377: 2044–2052. 29116890 10.1056/NEJMoa1705026 PMC5629452

[109] Ozoh GA , Murdoch ME , Bissek AC *et al* The African programme for onchocerciasis control: impact on onchocercal skin disease. Trop Med Int Health 2011: 16: 875–883. 21481109 10.1111/j.1365-3156.2011.02783.x

[110] King CH . Health metrics for helminth infections. Acta Trop 2015: 141: 150–160. 24333545 10.1016/j.actatropica.2013.12.001 PMC4055550

[111] King CH , Bertino AM . Asymmetries of poverty: why global burden of disease valuations underestimate the burden of neglected tropical diseases. PLoS Negl Trop Dis 2008: 2: e209. 18365036 10.1371/journal.pntd.0000209 PMC2267491

[112] Alonso LM , Murdoch ME , Jofre‐Bonet M . Psycho‐social and economical evaluation of onchocerciasis: a literature review. Soc Med 2009: 4: 8–31.

[113] Taylor HR , Jonas JB , Keeffe J *et al* Disability weights for vision disorders in Global Burden of Disease study. Lancet: 381: 23. 10.1016/S0140-6736(12)62081-9 23266227

[114] Salomon JA , Vos T , Murray CJL . Disability weights for vision disorders in Global Burden of Disease study ‐ Authors’ reply. Lancet 381: 23–24. 10.1016/S0140-6736(12)62131-X 23266228

[115] Nord E . Disability weights in the Global Burden of Disease 2010: unclear meaning and overstatement of international agreement. Health Policy 2013: 111: 99–104. 23608637 10.1016/j.healthpol.2013.03.019

[116] Colebunders R , Titulaer MJ . Nodding syndrome: preventable and treatable. Sci Transl Med 2017: 9: eaam8532. 28202778 10.1126/scitranslmed.aam8532

[117] Chesnais CB , Nana‐Djeunga HC , Njamnshi AK *et al* The temporal relationship between onchocerciasis and epilepsy: a population‐based cohort study. Lancet Infect Dis 2018: 18: 1278–1286. 30268645 10.1016/S1473-3099(18)30425-0

[118] Vinkeles Melchers NVS , Mollenkopf S , Colebunders R *et al* Burden of onchocerciasis‐associated epilepsy: first estimates and research priorities. Infect Dis Poverty 2018: 7: 101. 30253788 10.1186/s40249-018-0481-9 PMC6156959

[119] Krotneva SP , Coffeng LE , Noma M *et al* African Program for Onchocerciasis Control 1995–2010: impact of Annual Ivermectin Mass Treatment on Off‐Target Infectious Diseases. PLoS Negl Trop Dis 2015: 9: e0004051. 26401658 10.1371/journal.pntd.0004051 PMC4581698

[120] Walker M , Stolk WA , Dixon MA *et al* Modelling the elimination of river blindness using long‐term epidemiological and programmatic data from Mali and Senegal. Epidemics 2017: 18: 4–15. 28279455 10.1016/j.epidem.2017.02.005 PMC5340858

[121] Frempong KK , Walker M , Cheke RA *et al* Does Increasing Treatment Frequency Address Suboptimal Responses to Ivermectin for the Control and Elimination of River Blindness? Clin Infect Dis 2016: 62: 1338–1347. 27001801 10.1093/cid/ciw144 PMC4872292

[122] Doyle SR , Bourguinat C , Nana‐Djeunga HC *et al* Genome‐wide analysis of ivermectin response by Onchocerca volvulus reveals that genetic drift and soft selective sweeps contribute to loss of drug sensitivity. PLoS Negl Trop Dis 2017: 11: e0005816. 28746337 10.1371/journal.pntd.0005816 PMC5546710

[123] Husereau D , Drummond M , Petrou S *et al* Consolidated Health Economic Evaluation Reporting Standards (CHEERS) statement. BMJ 2013: 11: 346. 10.1136/bmj.f1049 23529982

[124] McFarland D , Murray J . A review of the economic impact of treatment with ivermectin for Onchocerciasis. Atlanta: Emory University School of Public Health and the International Health Program Office at the Centers for Disease Control and Prevention, 1994.

[125] Benton B . Economic impact of onchocerciasis control through the African Programme for Onchocerciasis Control: an overview. Ann Trop Med Parasitol 1998: 92(Suppl 1): S33–S39. 9861265 10.1080/00034989859537

[126] Prescott N , Prost A , Le Berre R . The economics of blindness prevention in Upper Volta under the Onchocerciasis Control Program. Soc Sci Med 1984: 19: 1051–1055. 6098020 10.1016/0277-9536(84)90307-1

[127] Prost A , Prescott N . Cost‐effectiveness of blindness prevention by the Onchocerciasis Control Programme in Upper Volta. Bull World Health Organ 1984: 62: 795–802. 6439428 PMC2536219

[128] Evans TG . Socioeconomic consequences of blinding onchocerciasis in west Africa. Bull World Health Organ 1995: 73: 495–506. 7554022 PMC2486790

[129] Kim A , Tandon A , Hailu A . Health and Labor Productivity: The Economic Impact of Onchocercial Skin Disease. Policy, Research working paper; no WPS 1836 Washington, DC: World Bank. 1997 (Available from: http://documentsworldbankorg/curated/en/417751468767965429/Health-and-labor-productivity-the-economic-impact-of-onchocercal-skin-disease).

[130] Okeibunor JC , Amuyunzu‐Nyamongo M , Onyeneho NG *et al* Where would I be without ivermectin? Capturing the benefits of community‐directed treatment with ivermectin in Africa. Trop Med Int Health 2011: 16: 608–621. 21395925 10.1111/j.1365-3156.2011.02735.x

[131] Oladepo O , Brieger WR , Otusanya S , Kale OO , Offiong S , Titiloye M . Farm land size and onchocerciasis status of peasant farmers in south‐western Nigeria. Trop Med Int Health 1997: 2: 334–340. 9171841 10.1111/j.1365-3156.1997.tb00148.x

[132] Thomson IG . Onchocerciasis in an oil palm estate. Trans R Soc Trop Med Hyg 1971: 65: 484–489. 5315409 10.1016/0035-9203(71)90158-1

[133] Wogu MD , Okaka CE . Prevalence and socio‐economic effects of onchocerciasis in Okpuje, Owan West Local Government Area, Edo State, Nigeria. Int J Biomed Health Sci 2008: 4: 113–119.

[134] Workneh W , Fletcher M , Olwit G . Onchocerciasis in field workers at Baya Farm, Teppi Coffee Plantation Project, southwestern Ethiopia: prevalence and impact on productivity. Acta Trop 1993: 54: 89–97. 7902651 10.1016/0001-706x(93)90054-f

[135] Frick KD , Foster A , Bah M , Faal H . Analysis of costs and benefits of the Gambian Eye Care Program. Arch Ophthalmol 2005: 123: 239–243. 15710822 10.1001/archopht.123.2.239

[136] Smith TS , Frick KD , Holden BA , Fricke TR , Naidoo KS . Potential lost productivity resulting from the global burden of uncorrected refractive error. Bull World Health Organ 2009: 87: 431–437. 19565121 10.2471/BLT.08.055673 PMC2686211

[137] Shamanna BR , Dandona L , Rao GN . Economic burden of blindness in India. Indian J Ophthalmol 1998: 46: 169–172. 10085631

[138] World Health Organization . Global burden of onchocerciasis in the year 2000: Summary of methods and data sources. 2006. (Available from:https://www.who.int/healthinfo/statistics/bod_onchocerciasis.pdf).

[139] World Health Organization . Global Burden of Disease 2004 update: Disability weights for diseases and conditions. 2008. (Available from: https://www.who.int/healthinfo/global_burden_disease/GBD2004_DisabilityWeights.pdf).

[140] Salomon JA , Vos T , Hogan DR *et al* Common values in assessing health outcomes from disease and injury: disability weights measurement study for the Global Burden of Disease Study 2010. Lancet 2012: 380: 2129–2143. 23245605 10.1016/S0140-6736(12)61680-8 PMC10782811

[141] de Vlas SJ , Stolk WA , le Rutte EA *et al* Concerted efforts to control or eliminate neglected tropical diseases: how much health will be gained? PLoS Negl Trop Dis 2016: 10: e0004386. 26890362 10.1371/journal.pntd.0004386 PMC4758649

[142] Remme J , Dadzie KY , Rolland A , Thylefors B . Ocular onchocerciasis and intensity of infection in the community. I. West African savanna. Trop Med Parasitol 1989: 40: 340–347. 2617045

[143] Remme JHF . The Global Burden of Onchocerciasis in 1990. Geneva: WHO, 2004. (Available from: https://www.who.int/healthinfo/global_burden_disease/Onchocerciasis%201990.pdf).

[144] Brown R , Shannon R . Prevalence, intensity and ocular manifestations of Onchocerca volvulus infection in Dimbelenge, Zaire. Ann Soc Belg Med Trop 1989: 69: 137–142. 2802810

[145] Henry MC , Meredith SE . The onchocerciasis focus at Kinsuka/Kinshasa (Republic of Zaire) in 1985. I. Entomological aspect. Ann Trop Med Parasitol 1990: 84: 369–379. 2260901 10.1080/00034983.1990.11812482

[146] Whitworth JAG , Morgan D , Gilbert CE , Mabey DM , Maude GH , Taylor DW . Effects of repeated doses of ivermectin on ocular onchocerciasis: community‐based trial in Sierra Leone. Lancet 1991: 338: 1100–1103. 1682543 10.1016/0140-6736(91)91963-u

[147] Whitworth JA , Gilbert CE , Mabey DM , Morgan D , Foster A . Visual loss in an onchocerciasis endemic community in Sierra Leone. Br J Ophthalmol 1993: 77: 30–32. 8435395 10.1136/bjo.77.1.30 PMC504418

[148] Kayembe DL , Kasonga DL , Kayembe PK , Mwanza JC , Boussinesq M . Profile of eye lesions and vision loss: a cross‐sectional study in Lusambo, a forest‐savanna area hyperendemic for onchocerciasis in the Democratic Republic of Congo. Trop Med Int Health 2003: 8: 83–89. 12535256 10.1046/j.1365-3156.2003.00957.x

[149] Fobi G , Yameogo L , Noma M *et al* Managing the Fight against Onchocerciasis in Africa: APOC Experience. PLoS Negl Trop Dis 2015: 9: e0003542. 25974211 10.1371/journal.pntd.0003542 PMC4431813

[150] Homeida M , Braide E , Elhassan E *et al* APOC's strategy of community‐directed treatment with ivermectin (CDTI) and its potential for providing additional health services to the poorest populations. African Programme for Onchocerciasis Control. Ann Trop Med Parasitol 2002: 96(Suppl 1): S93–S104. 12081254 10.1179/000349802125000673

[151] World Health Organization . African Programme for Onchocerciasis Control (APOC): About us. (Available from: http://www.who.int/apoc/about/en/).

[152] World Health Organization . Progress towards eliminating onchocerciasis in the WHO Region of the Americas: verification of elimination of transmission in Guatemala. Wkly Epidemiol Rec 2016: 91: 501–505. 27801556

[153] Torgerson DJ , Raftery J . Discounting. BMJ 1999: 319: 914–915. 10506056 10.1136/bmj.319.7214.914 PMC1116731

[154] Sheldon TA . Discounting in health care decision‐making: time for a change? J Public Health Med 1992: 14: 250–256. 1419202

[155] Attema AE , Brouwer WBF , Claxton K . Discounting in economic evaluations. Pharmacoeconomics 2018: 36: 745–758. 29779120 10.1007/s40273-018-0672-z PMC5999124

